# Maturation of human cardiac organoids enables complex disease modeling and drug discovery

**DOI:** 10.1038/s44161-025-00669-3

**Published:** 2025-06-25

**Authors:** Mark W. Pocock, Janice D. Reid, Harley R. Robinson, Natalie Charitakis, James R. Krycer, Simon R. Foster, Rebecca L. Fitzsimmons, Mary Lor, Lynn A. C. Devilée, Christopher A. P. Batho, Natasha Tuano, Sara E. Howden, Katerina Vlahos, Kevin I. Watt, Adam T. Piers, Kaitlyn Bibby, James W. McNamara, Rebecca Sutton, Valerii Iaprintsev, Jacob Mathew, Holly K. Voges, Patrick R. J. Fortuna, Sebastian Bass-Stringer, Celine Vivien, James Rae, Robert G. Parton, Anthony B. Firulli, Leszek Lisowski, Hannah Huckstep, Sean J. Humphrey, Sean Lal, Igor E. Konstantinov, Robert G. Weintraub, David A. Elliott, Mirana Ramialison, Enzo R. Porrello, Richard J. Mills, James E. Hudson

**Affiliations:** 1 QIMR Berghofer, Brisbane, Queensland Australia; 2 School of Biomedical Sciences, Queensland University of Technology, Brisbane, Queensland Australia; 3 School of Biomedical Sciences, Faculty of Medicine, The University of Queensland, Brisbane, Queensland Australia; 4 Murdoch Children’s Research Institute, The Royal Children’s Hospital, Melbourne, Victoria Australia; 5 Novo Nordisk Foundation Center for Stem Cell Medicine, Murdoch Children’s Research Institute, Melbourne, Victoria Australia; 6 Department of Paediatrics, University of Melbourne, Melbourne, Victoria Australia; 7 Department of Pharmacology, Monash University, Melbourne, Victoria Australia; 8 Department of Anatomy & Physiology, School of Biomedical Sciences, The University of Melbourne, Melbourne, Victoria Australia; 9 Melbourne Centre for Cardiovascular Genomics and Regenerative Medicine, Melbourne, Victoria Australia; 10 Department of Cardiology, Royal Children’s Hospital, Melbourne, Victoria Australia; 11 Institute for Molecular Bioscience, The University of Queensland, Brisbane, Queensland Australia; 12 Centre for Microscopy and Microanalysis, The University of Queensland, Brisbane, Queensland Australia; 13 Herman B Wells Center for Pediatric Research, Department of Pediatrics, Anatomy, Biochemistry, and Medical and Molecular Genetics, Indiana University School of Medicine, Indianapolis, IN USA; 14 Translational Vectorology Research Unit, Children’s Medical Research Institute, Faculty of Medicine and Health, The University of Sydney, Sydney, New South Wales Australia; 15 Laboratory of Molecular Oncology and Innovative Therapies, Military Institute of Medicine - National Research Institute, Warsaw, Poland; 16 School of Medical Sciences, Faculty of Medicine and Health, University of Sydney, Sydney, New South Wales Australia; 17 Department of Cardiothoracic Surgery, Royal Children’s Hospital, Melbourne, Victoria Australia; 18 Australian Regenerative Medicine Institute, Monash University, Melbourne, Victoria Australia

**Keywords:** Tissue engineering, Differentiation, Cardiomyopathies

## Abstract

Maturation of human pluripotent stem (hPS) cell-derived cardiomyocytes is critical for their use as a model system. Here we mimic human heart maturation pathways in the setting of hPS cell-derived cardiac organoids (hCOs). Specifically, transient activation of 5′ AMP-activated protein kinase and estrogen-related receptor enhanced cardiomyocyte maturation, inducing expression of mature sarcomeric and oxidative phosphorylation proteins, and increasing metabolic capacity. hCOs generated using the directed maturation protocol (DM-hCOs) recapitulate cardiac drug responses and, when derived from calsequestrin 2 (*CASQ2*) and ryanodine receptor 2 (*RYR2*) mutant hPS cells exhibit a pro-arrhythmia phenotype. These DM-hCOs also comprise multiple cell types, which we characterize and benchmark to the human heart. Modeling of cardiomyopathy caused by a desmoplakin (*DSP*) mutation resulted in fibrosis and cardiac dysfunction and led to identifying the bromodomain and extra-terminal inhibitor INCB054329 as a drug mitigating the desmoplakin-related functional defect. These findings establish DM-hCOs as a versatile platform for applications in cardiac biology, disease and drug screening.

## Abstract (web summary)

Pocock et al. reveal that transient activation of 5′ AMP-activated protein kinase and estrogen-related receptor drives robust maturation of multicellular human cardiac organoids, enabling modeling of desmoplakin cardiomyopathy dysfunction, which could be rescued using the bromodomain and extra-terminal inhibitor INCB054329.

## Main

Understanding maturation is pivotal for many fields, including the use of hPS cell technologies for disease modeling and drug discovery. Recent studies have begun to identify factors required for maturation^1,2^, but it has been difficult to pinpoint the upstream stimuli sufficient to drive maturation. hPS cell-derived cardiomyocytes are typically immature, limiting their applications for disease modeling and drug screening. Extended culture for even 1 year does not result in equivalent time-matched in vivo maturation^3^, indicating that either culture conditions are inhibitory and/or the critical stimuli are absent. Different stimuli drive distinct aspects of maturation^4^, and the most successful drivers of maturation thus far are multicellularity, mechanical loading/structural patterning, hormonal stimulation, metabolic switching and pacing with electrical stimulation^1^. Multicellular mixing and organoid protocols have been established to promote cellular complexity^5–9^. Tissue engineering approaches generally incorporate mechanical loading, which improves the morphology, alignment and maturity^4,10–12^. Fatty acid metabolism promotes metabolic maturation, cell cycle shut down and expression of mature sarcomeric isoforms^4^. Pacing is one of the most potent inducers of functional maturation in terms of excitation–contraction coupling and drug responses^13–15^. Despite these advances, mechanistic understanding is limited, and the induction of further maturation is challenging.

Heart-Dyno is a 96-well platform that facilitates the self-organization of cardiac cell types into miniaturized, mechanically loaded hCOs^4,8^. To create hCOs, hPS cells are carefully patterned into pre-cardiac mesoderm^16^, which gives rise to cardiomyocytes and cardiac progenitor cells in a single directed differentiation protocol. These cells are used to fabricate multicellular hCOs^8^, which we mature using metabolic switching to oxidative phosphorylation^4^. Yet, consistent with all current protocols, adult cardiac properties remain elusive. In this study we use our knowledge of human heart maturation^17^ to screen for conditions that drive maturation. This revealed that transient activation of 5′ AMP-activated protein kinase (AMPK) and estrogen-related receptor (ERR) induces robust maturation manifested in maturation of the transcriptome, proteome, contractile function and metabolic capacity. Using extensive characterization, drug testing and disease modeling experiments, we demonstrate that these DM-hCOs are a faithful model system able to model complex diseases.

## Results

### hPS cell-derived cardiomyocyte platforms require further maturation

**Extended Data Fig. 1:**
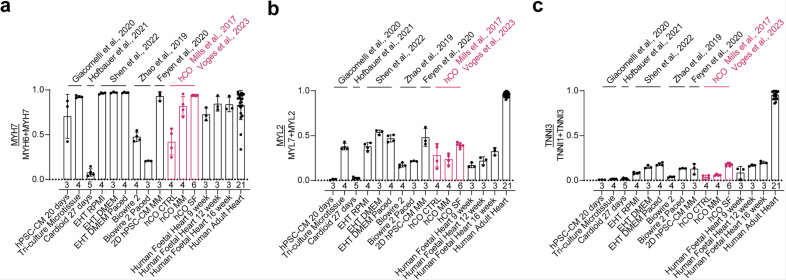
Comparison of mRNA expression of sarcomeric protein ratios that correlate with maturation across multiple human pluripotent stem cell derived cardiomyocyte platforms. **a**, Fraction of *MYH7*. **b**, Fraction of *MYL2*. **c**, Fraction of *TNNI3*. Gene counts expressed as a ratio. All n are biological. Data are mean ± standard deviation. RNA-sequencing data collected from hPSC-CM cultures including GSE93841, GSE148025, GSE116464, GSE201437, GSE114976, GSE151279 and human hearts including ERP109940 and Hahn et al., 2021. Source data

We compared our most recent serum-free hCO protocol (SF-hCOs)^8^ to hPS cell-derived cardiomyocyte cultures using ratios of sarcomeric isoforms that are indicative of maturation^4,7,9,13,15,18–20^. *MYH7* as a fraction of *MYH7* and *MYH6*, did not correlate with maturation, and seems to be rate dependent (Extended Data Fig. 1a). *MYL2* as a fraction of *MYL2* and *MYL7* (Extended Data Fig. 1b) and *TNNI3* as a fraction of *TNNI3* and *TNNI1* (Extended Data Fig. 1c) were strong indicators of maturation, particularly in pacing protocols and metabolic maturation conditions. However, the highest *TNNI3* fraction was only 18%, which is equivalent to a ~20-week fetal heart. Our SF-hCOs go through a metabolic maturation phase^4,21^, display a similar level of maturation to the pacing protocols and provide a mid-gestational stage to screen for maturation (Extended Data Fig. 1c). Given the expression of the mature sarcomeric isoform *TNNI3* is consistently low (Extended Data Fig. 1c), we used staining of cardiac troponin I (cTnI, encoded by *TNNI3*) as a primary readout of maturation^3^. In the final screening protocols, we assessed function following removal of the stimuli for at least 2 days. This enables the assessment of whether there is reduced automaticity (without impacting force or substantially increasing the time to 50% relaxation (Tr50)), which is another key feature of maturation.

### AMPK and ERR agonists drive maturation

**Fig. 1:**
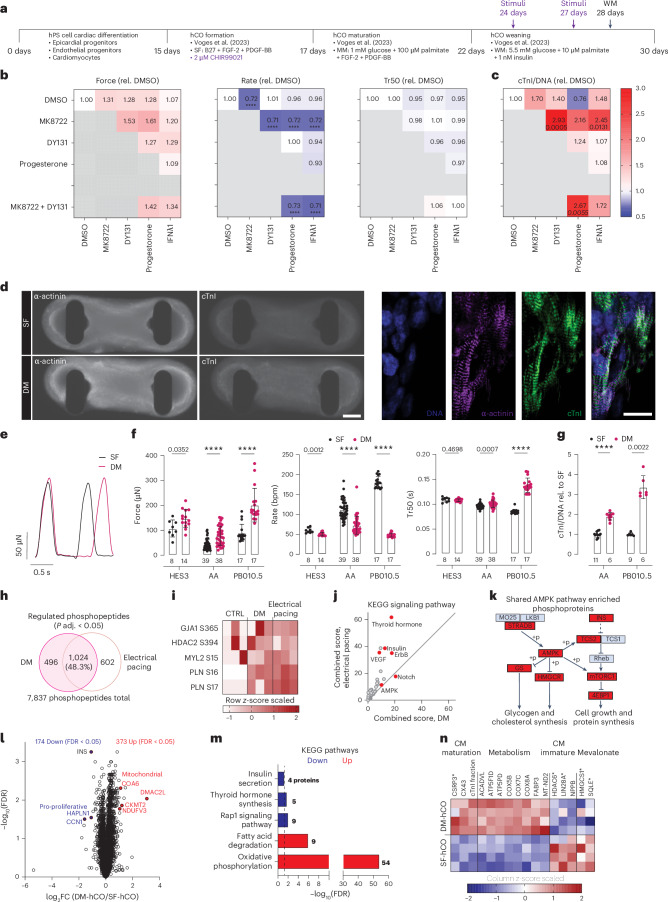
Screening for combinatorial stimuli to promote hCO maturation. **a**, Schematic of the protocol. **b**, Force of contraction, rate and Tr50 at 30 days normalized to pretreatment at 24 days. *n* = 11–17 hCOs from three experiments. **c**, cTnI intensity normalized to DNA and then to dimethylsulfoxide (DMSO) controls. *n* = 9–14 hCOs from two experiments. **d**, cTnI staining of cardiomyocytes (α-actinin). Scale bars, 200 μm (left) and 20 μm (right). Representative of data from three cell lines. **e**, Comparison of SF-hCO and DM-hCO raw contraction curves. **f**, Comparison of raw force of contraction, rate and Tr50 between SF-hCOs and DM-hCOs for three different cell lines (HES3, AA and PB010.5). *n* = hCOs. **g**, cTnI intensity normalized to DNA and then to DMSO controls for two different cell lines (AA and PB010.5). *n* = hCOs. **h**, Differentially regulated phosphosites in MK8722 + DY131 (DM)-treated and 120-bpm electrically paced hCOs (5-min stimulation each), highlighting shared sites. *n* = 3 biological replicates with 15 pooled hCOs each. **i**, Heat map of phosphosite *z*-scores. **j**, Gene Ontology analysis of phosphorylated proteins shared between acute DM treatment of hCOs and electrical pacing. **k**, The AMPK signaling network shared between acute DM treatment of hCOs and electrical pacing. **l**, Volcano plot of DM-hCO versus SF-hCO proteomics abundance data. **m**, Gene Ontology analysis of differentially regulated proteins in DM-hCOs versus SF-hCOs. *n* = 4 biological replicates with three pooled hCOs each. **n**, Heat map of selected significantly differentially regulated (FDR < 0.05) protein expression *z*-scores with asterisks indicating significance by *P* value only (*P* < 0.05). cTnI fraction was calculated as cTnI / (cTnI + ssTnI) in each sample. Concentrations: 10 μM progesterone, 3 μM DY131, 10 μM MY8722 and 100 ng ml^−1^ IFNλ1. Data are the mean ± s.d. Kruskal–Wallis test with Dunn’s multiple comparison to DMSO (**b** and **c**) and two-tailed Mann–Whitney tests for each cell line (**f** and **g**) were performed. In the heat maps (**b** and **c**), the *P* values are shown beneath the values if significant. ****P < 0.0001. CM, cardiomyocyte; FC, fold change; KEGG, Kyoto Encyclopedia of Genes and Genomes. Source data

Transcriptional profiling of human heart maturation has identified a key oxidative metabolism and interferon signature that strongly underpins cardiomyocyte maturation^17^. We cannot electrically pace hCOs in the Heart-Dyno for extended periods of time without causing toxicity. Initially, multiple strategies to increase rate and metabolism using isoprenaline, optogenetic pacing^22^ or pacemaker cells were tested but were ineffective in substantially inducing increased rate (Supplementary Figs. 1–3). In an alternative pharmacological approach, we screened for factors that increase metabolism and interferon signaling to promote maturation (Supplementary Figs. 4–7). This identified 10 μM progesterone, 3 μM DY131 (ERRβ/γ agonist), 10 μM MK8722 (AMPK activator) and 100 ng ml^−1^ IFNλ1 as potentially beneficial, which were then assessed in combinatorial screening (Fig. 1a). Conditions with MK8722 consistently reduced rate without impacting Tr50 (Fig. 1b). MK8722 combined with DY131 or IFNλ1 increased cTnI (Fig. 1c). However, when MK8722 and DY131 were further combined with either progesterone or IFNλ1, there was no additional increase in cTnI (Fig. 1c). The timing of DY131 and MK8722 addition was also assessed (Supplementary Fig. 8). If added during the maturation medium period (days 17–22) or immediately after this period at the start of the weaning medium period (days 22–27), rate was still reduced but cTnI increase was limited. Different fatty acid types (palmitate, linoleate, oleate and myristylate) performed equally well, so we continued to use palmitate in our protocol (Supplementary Fig. 9). The DM-hCO protocol incorporates these key additions to the SF-hCO protocol^8^: the addition of 2 μM CHIR99021 during the first 2 days of hCO formation and transient 4-day addition of 3 μM DY131 combined with 10 μM MK8722 (days 24–28). The DM-hCO protocol was consistently able to reduce rate and increase cTnI and force in multiple additional hPS cell lines (Fig. 1d–g). To determine whether these conditions are broadly applicable to the field, we assessed the impact of our protocol in a two-dimensional (2D) culture. The addition of MK8722 during a 4-day period (days 24–28) was also sufficient to increase cTnI in 2D hPS cell-derived cardiomyocytes (Supplementary Fig. 10).

As there is a close relationship between exercise, AMPK and metabolism in the heart^23^, the similarity in the activation profile of acute pacing and DM treatment as assessed using phosphoproteomics (Supplementary Fig. 11). We added Heart-Dyno inserts into a C-pace system in 24-well plates enabling 120-bpm pacing for 5 min without causing toxicity. Phosphoproteomics revealed that there was substantial overlap of 1,024 (48.2% of differentially regulated) phosphosites of DM treatment and electrical pacing (Fig. 1h). Phosphosites known to play a role in cardiac biology and function were detected including HDAC2 S394 (ref. ^24^), GJA1 (CX43) S365 (ref. ^25^), PLN S16/T17 and MYL2 (MLC2v) S15 (ref. ^26^). PLN S16/T17 consistently increased in both acutely DM-treated and paced hCOs and increased sarcoplasmic reticulum calcium cycling^27^ (Fig. 1i). Similar Kyoto Encyclopedia of Genes and Genomes pathways were activated in both DM and electrically paced conditions (Fig. 1j), including an AMPK network (Fig. 1k) and inhibition of mevalonate/cholesterol biosynthesis, which we have shown to decrease during cardiac maturation^28^.

Proteomics was used to compare global protein changes in DM-hCOs versus SF-hCOs (Fig. 1l–n). There was an increase in cTnI fraction confirming the screening results (Fig. 1n). Consistent with phosphoproteomics, there was also repression of immature signatures including insulin signaling and the mevalonate pathway^28^. However, the response was dominated by upregulation of metabolic proteins, consistent with the metabolic changes during cardiac maturation^17^ (Fig. 1l–n).

### hCOs display cellular complexity similar to human hearts

**Fig. 2:**
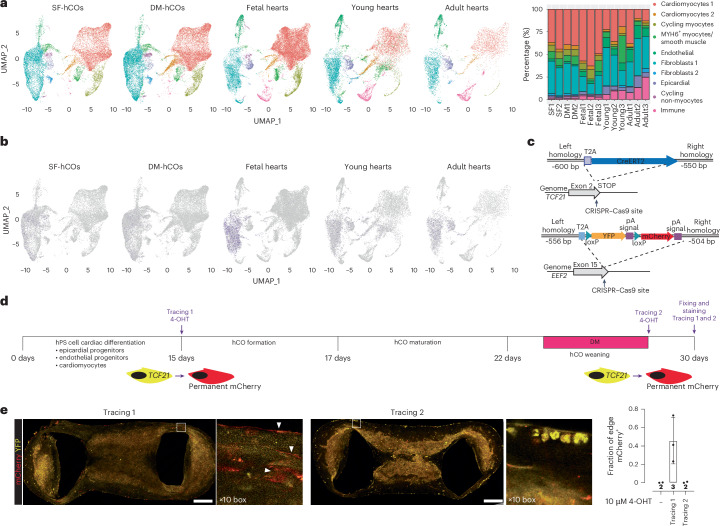
Cellular composition in SF-hCOs and DM-hCOs. **a**, Co-clustering of snRNA-seq from SF-hCOs and DM-hCOs and human heart data from GSE156707 (ref. ^17^), including proportions of cell populations. *n* = 2 snRNA-seq biological replicates for SF-hCOs and DM-hCOs, each containing ~70 pooled hCOs. **b**, Expression of pro-epicardial organ marker *TCF21*. **c**, Generation of a hPS cell *TCF21* lineage tracing line. **d**, Schematic of lineage tracing experiments. **e**, Representative analysis of lineage tracing 1 and 2 along with quantification of hCO coverage across multiple experiments. *n* = 2–3 experiments. Data are the mean ± s.d. White arrowheads indicate epicardial cells on the surface and fibroblasts that are integrated within the hCO tissue. Scale bars, 200 μm. 4-OHT, 4-hydroxytamoxifen. Source data

To dissect the cell-specific maturation responses, we used single-nuclei RNA sequencing (snRNA-seq) to compare SF-hCOs and DM-hCOs. The SF-hCO and DM-hCO cells all co-cluster with multiple in vivo cardiac cell types^17^, including cardiomyocytes, endothelial cells, smooth muscle cells, fibroblasts and epicardial cells (Fig. 2a), yielding a complex mixture of cells similar to native human heart tissue^17^. However, there is a higher percentage of cardiomyocytes, lower percentage of endothelial cells, lack of immune cell populations (Fig. 2a) and lack of a neural population at higher clustering resolution (data not shown) in hCOs. To explore fibroblast identity, the epicardial gene *TCF21* was expressed at low levels, indicative of epicardial origins (Fig. 2b). As *TCF21* decreases following differentiation^29^, we created a *TCF21* lineage tracing tool (Fig. 2c). Early, but not late, lineage tracing marked the epicardial cells surrounding the SF-hCOs and DM-hCOs and infiltrating cells (Fig. 2d,e). Thus, hCOs contain multicellular populations similar to the human heart.

### hCOs display complex cell–cell interactions

**Fig. 3:**
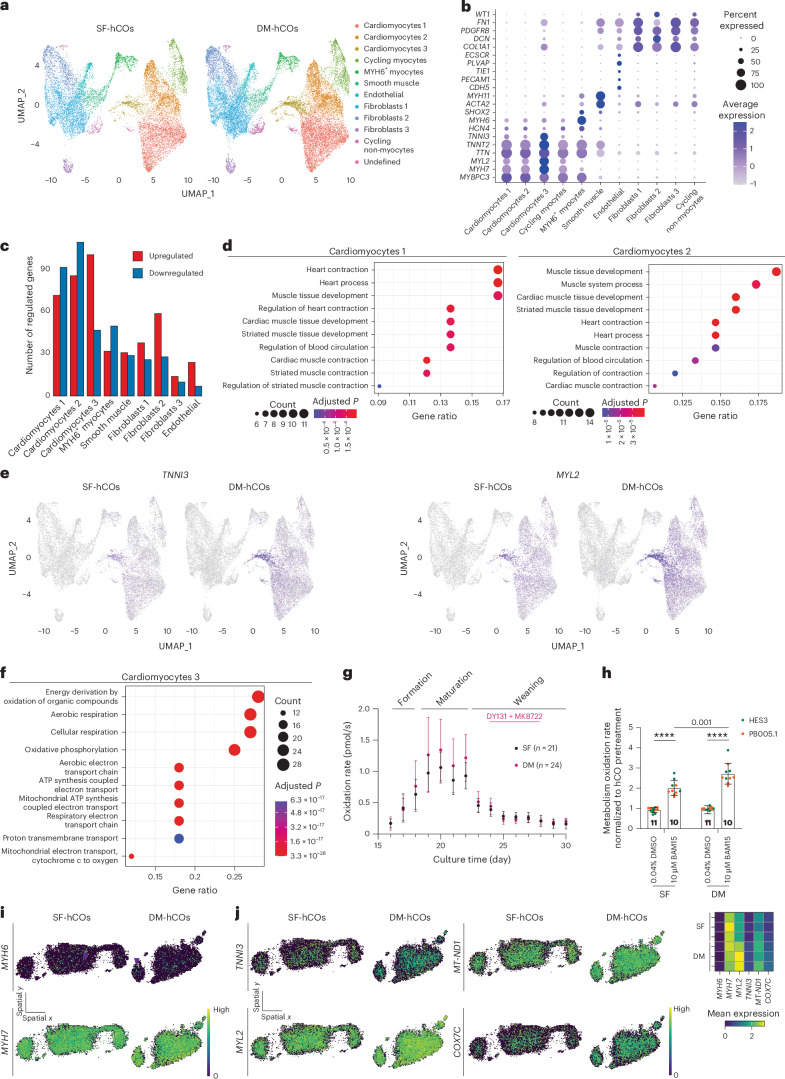
Sarcomeric and metabolic maturation in DM-hCOs. **a**, Uniform manifold approximation and projection (UMAP) of nuclei in SF-hCOs and DM-hCOs. Nuclei are labeled by cell type. **b**, Expression of canonical cell markers in SF-hCOs and DM-hCOs. **c**, Number of differentially regulated genes in different cellular populations from DM-hCOs versus SF-hCOs using pseudo-bulk analysis (average log_2_FC > |0.25|, adjusted *P* < 0.05). **d**, Top ten enriched Gene Ontology terms from the sub-ontology ‘biological processes’ for upregulated genes in cardiomyocyte 1 and 2 populations. **e**, Expression of sarcomeric maturation genes *TNNI3* and *MYL2*. **f**, Top ten enriched Gene Ontology terms from the sub-ontology ‘biological processes’ for upregulated genes in the cardiomyocyte 3 cluster. **g**, Oxidation rates in SF-hCOs and DM-hCOs over the culture duration. *n* = hCOs from two cell lines (HES3 and PB005.1). **h**, Response to BAM15 in SF-hCOs and DM-hCOs. *n* = hCOs from two cell lines (HES3 and PB005.1). **i**, Representative spatial expression of cardiomyocyte (*MYH7*) and nodal cardiomyocyte (*MYH6*) markers in a section. Purple arrowheads indicate *MYH6* clusters. **j**, Representative spatial expression of sarcomeric and metabolic genes in a section, including a heat map of average expression. Data are the mean ± standard deviation (**g** and **h**). Two-way analysis of variance with Tukey’s multiple-comparison test (**h**) was performed. ****P < 0.0001. Source data

**Extended Data Fig. 2:**
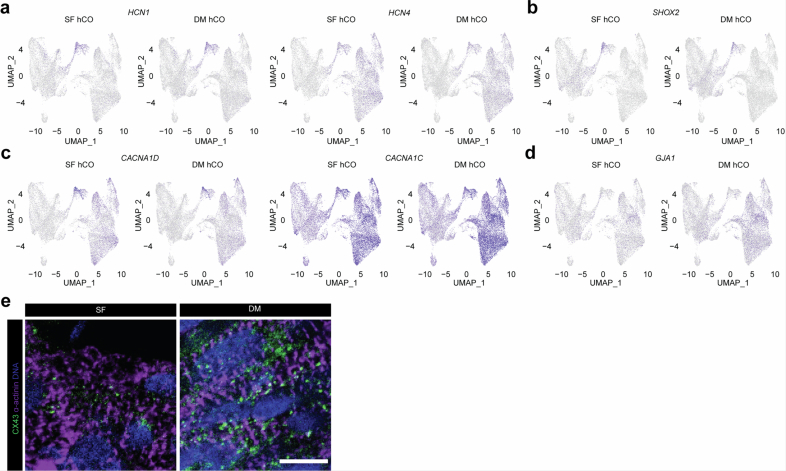
Critical pace controlling genes in both SF- and DM-hCOs. **a**, *I*_*f*_ channel genes *HCN1* and *HCN4*. **b**, A key nodal cell transcription factor, *SHOX2*. **c**, Calcium channel genes for Cav1.2 (*CACNA1C*) and Cav1.3 (*CACNA1D*). **d**, CX43 cell-cell junction gene *GJA1*. **e**, Immunostaining of CX43 in SF versus DM-hCOs. Representative of hCOs from 3 different cell lines. Scale bar = 10 μm.

The cellular composition is unaltered in DM-hCOs versus SF-hCOs, and immunostaining confirmed the presence of the major cardiac cell types including cardiomyocytes, epicardial cells, endothelial cells, pericytes and fibroblasts (Fig. 2a and Supplementary Fig. 12). When unsupervised dimensionality reduction and clustering analysis was performed on hCO nuclei alone, this also revealed distinct cellular populations (Fig. 3a and Supplementary Fig. 13), which are demarcated by canonical marker genes (Fig. 3b). The SF-hCO and DM-hCO *MYH6*^+^ cardiomyocytes express critical ‘funny’ current (*I*_*f*_) channel genes *HCN1* and *HCN4* and a critical pacemaker transcription factor encoded by *SHOX2* (ref. ^30^; Extended Data Fig. 2a,b). This population likely represents the pacemaker population within the SF-hCOs and DM-hCOs, which we previously confirmed using patch clamping^4^.

**Extended Data Fig. 3:**
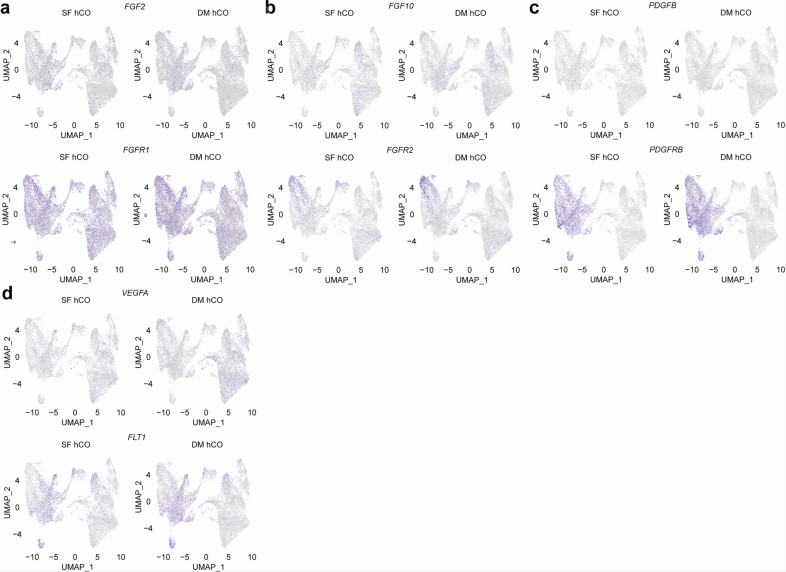
Paracrine interactions in SF- and DM-hCOs. **a**, *FGF2-FGFR1*. **b**, *FGF10-FGFR2*. **c**, *PDGFB-PDGFRB*. **d**, *VEGFA-FLT1*.

In our protocol, exogenous growth factors FGF-2 and PDGF-BB are only required during the first week of hCO culture. Extended addition or incorporation of more cardiac fibroblasts results in fibroblast overpopulation and uncoordinated contractions in hCOs^8^. This suggests that maintenance of the fibroblasts and endothelial cells occurs via endogenous production of growth factors. Consistent with paracrine support of fibroblasts, there is low but ubiquitous expression of *FGF2* and its corresponding receptor *FGFR1* (Extended Data Fig. 3a), and cardiomyocytes and smooth muscle also express *FGF10*, the cognate ligand for the fibroblast receptor *FGFR2* (Extended Data Fig. 3b). While *PDGFRB* was abundantly expressed by the fibroblasts, the ligand *PDGFB* was expressed at very low levels (Extended Data Fig. 3c), indicating that fibroblast support may occur primarily via FGF signaling. Consistent with paracrine support of endothelial cells, cardiomyocytes express *VEGFA* and endothelial cells and some fibroblasts express the receptor *FLT1* (Extended Data Fig. 3d). Together, paracrine factors likely support hCO culture in the absence of exogenous growth factors.

### hCO cell subtypes are consistent with heart subtypes

**Extended Data Fig. 4:**
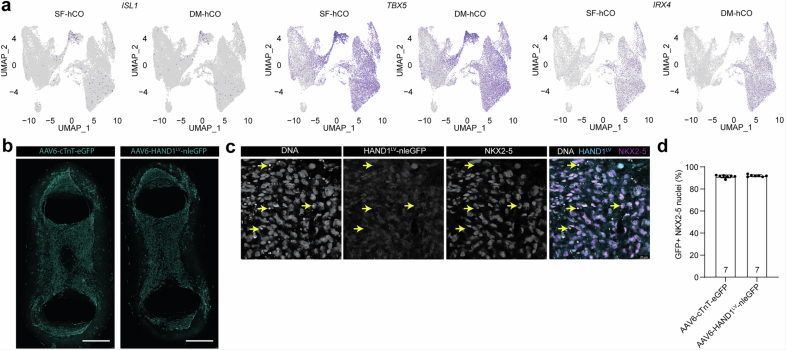
Cardiomyocyte sub-type. **a**, UMAP projection of nuclei in SF- and DM-hCOs for right side/outflow tract marker *ISL1*, left ventricle marker *TBX5* and ventricular marker *IRX4*. **b**, Overview of whole hCO expression of AAV6-cTnT-eGFP and AAV6-*Hand1*^*LV*^*-*nleGFP in SF-hCOs. Scale bars = 100 μm. **c**, Expression of AAV6-*Hand1*^*LV*^*-*nleGFP in predominately in the NKX2-5^+^ cardiomyocytes rather than the stromal cells in SF-hCOs. Arrows indicate NKX2-5^−^ stromal cells lacking AAV6-*Hand1*^*LV*^*-*nleGFP expression. Scale bar = 20 μm. **d**, Quantification of NKX2-5^+^ cardiomyocytes expressing eGFP in SF-hCOs. Data are mean ± standard deviation. Images in **b**, **c** are representative of the n = 7 hCOs in **d**. Source data

The subtypes of cardiomyocyte, fibroblast and endothelial populations were assessed in more detail. Using subtype markers^31^, the expression of the ventricular marker *IRX4* and left ventricle marker *TBX5*, together with the lack of expression of the right ventricular/outflow tract marker *ISL1 (*only expressed in the *MYH6*^+^ cluster), is indicative of a left ventricular subtype (Extended Data Fig. 4a). To validate this, we used a reporter driven by a *Hand1* enhancer, which is expressed in the left ventricle^32^. When delivered using adeno-associated virus serotype 6 (AAV6), this reporter marked nearly all the cardiomyocytes in hCOs using AAV6-cTnT-eGFP as a control (Extended Data Fig. 4b–d). For fibroblasts, canonical markers of pathological fibroblast activation including *ACTA2*, *FN1* and *POSTN* were not widely expressed in hCOs (Supplementary Fig. 14). For endothelial cells, endocardial markers *NRG1*, *NFATC1* and *GATA4* were highly expressed, whereas coronary markers *APLN* and *FABP4* were not (Supplementary Fig. 15), indicative of endocardial cell subtypes consistent with our recent findings^8^.

### DM-hCOs display advanced sarcomeric and metabolic maturation

Co-clustering of SF-hCO and DM-hCO cardiomyocytes with in vivo hearts yielded no drastic changes in cardiomyocyte subclusters (Supplementary Fig. 16). Differential gene expression analysis comparing adult hearts versus SF-hCOs for the most populous cardiomyocyte cluster (cardiomyocytes 1; Fig. 2a) revealed only 185 or 97 transcripts were twofold lower or higher, respectively (Supplementary Table 1). The DM-hCO protocol increases 20 (11%) and decreases 37 (38%) of these transcripts >log_2_|0.2| toward adult heart expression, respectively, indicating a maturation response.

When focusing only on hCO cell clusters (Fig. 3a,b), the largest transcriptional changes in DM-hCOs versus SF-hCOs were in cardiomyocytes and fibroblasts, accounting for 59% and 20% of all differentially expressed genes, respectively (Fig. 3c and Supplementary Table 2). Because the cardiomyocytes exhibited the largest number of differentially regulated genes, we explored these in further detail. *TNNI3* as a fraction of *TNNI3* plus *TNNI1* increased from 0.34, 0.25 and 0.23 in SF-hCOs to 0.44, 0.37 and 0.34 in cardiomyocyte clusters 1, 2 and 3, respectively. Genes upregulated in DM-hCO compared to SF-hCO cardiomyocyte clusters 1 and 2 were enriched in heart contraction and development ontologies (Fig. 3d). This includes sarcomeric maturation markers *TNNI3* and *MYL2* (Fig. 3e) and transcriptional regulators that control heart development, including *FOXP1* (ref. ^33^), *SOX6* (ref. ^34^) and *CSRP3* (ref. ^35^). Genes upregulated in DM-hCOs compared to SF-hCOs in cardiomyocyte cluster 3 were enriched for Gene Ontology terms mostly associated with oxidative phosphorylation (Fig. 3f). This includes direct ERRα/ERRγ target genes^36^ particularly those involved in mitochondrial electron transport (*COX5A*, *COX5B*, *COX6A1*, *COX7C*, *COX8A* and *CYCS*) and ATP synthesis (*ATP5F1D*, *ATP5F1B*, *ATP5PO*, *ATP5MC1*, *ATP5PD*, *ATP5MC3* and *ATP5PF*). Real-time monitoring of hCO respiration revealed similar respiration in SF-hCO and DM-hCO protocols, which peaked during the maturation medium phase (Fig. 3g). At the end of the protocol, maximal respiratory capacity was higher in DM-hCOs versus SF-hCOs, as assessed by treatment with the mitochondrial uncoupler BAM15 (Fig. 3h). Together, these findings highlight the enhanced transcriptional and metabolic maturation in DM-hCOs.

To assess whether maturation occurs homogenously, spatial transcriptomics was performed. We identified 953 genes, which were mostly cardiomyocyte transcripts. The cardiomyocytes were spread throughout the SF-hCOs and DM-hCOs (*MYH7*), and *MYH6*^+^ pacemaker cardiomyocytes were more sporadic with some clustered regions (Fig. 3i). DM-hCOs had uniform induction of the sarcomeric maturation markers *MYL2* and *TNNI3* and metabolic genes *MTND1P1* and *COX7C* (Fig. 3j). This highlights that maturation is homogenous in DM-hCOs.

While the DM-hCO cardiomyocytes had the most differentially regulated transcripts, there were also regulated transcripts in the stromal cells (Fig. 3b). Some of these are also fibroblast-subtype specific such as *COL15A1*, *COL19A1* and *TGFBR3*, which may play a key role in extracellular matrix biology and paracrine interactions (Supplementary Fig. 17).

### Decreased automaticity is multifactorial in DM-hCOs

**Fig. 4:**
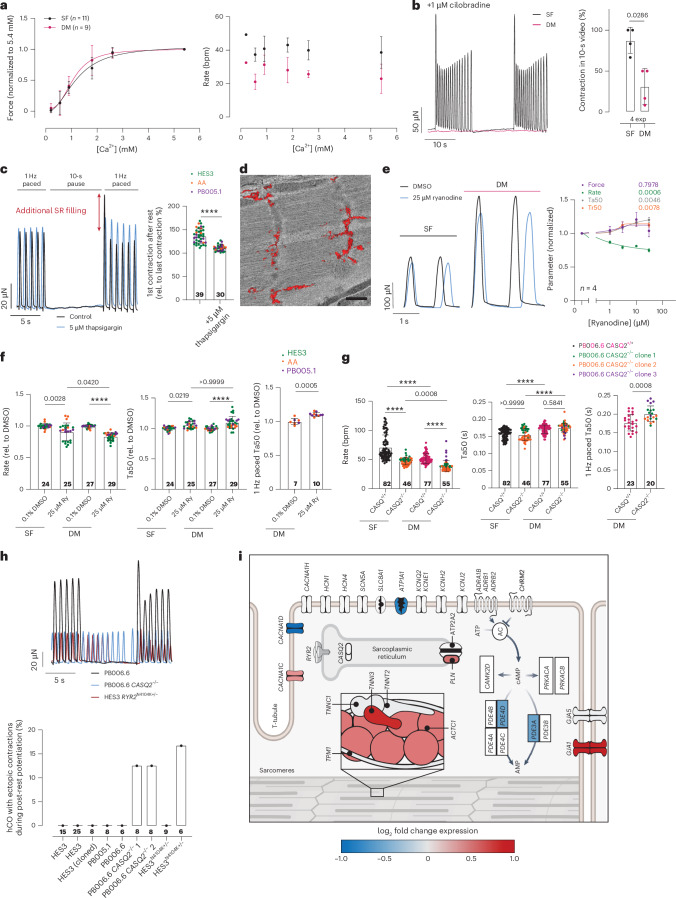
Mechanisms of rate control and sarcoplasmic reticulum handling in SF-hCOs and DM-hCOs. **a**, Dependence of force and rate on extracellular calcium concentration. *n* = hCOs from two experiments. **b**, Contractile rate/burst behavior with blockade of *I*_f_ using 1 μM cilobradine. *n* = 4 experiments. **c**, Post-rest-potentiation assessment of sarcoplasmic reticulum (SR) loading and verification using blockade of SERCA using 5 μM thapsigargin. *n* = hCOs pooled from three different lines (HES3, AA and PB005.1). **d**, Three-dimensional transmission electron microscopy rendering of the sarcoplasmic reticulum (red). Scale bar, 500 nm. **e**, Concentration–response curve of DM-hCOs treated with ryanodine and representative trace curves. Richard’s 5 parameter dose–response was used to fit the curves. *n* = hCOs. **f**, Influence of ryanodine on rate and Ta50, including under 1-Hz pacing for DM-hCOs. *n* = hCOs pooled from three different lines (HES3, AA and PB005.1). **g**, Influence of *CASQ2* knockout on rate and Ta50, including under 1-Hz pacing for DM-hCOs. *n* = hCOs pooled from three experiments for *CASQ2*^+/+^ or three different clones for *CASQ2*^−/−^. **h**, Quantification of ectopic contractions during the pause phase of post-rest-potentiation experiments. *n* = hCOs. **i**, Critical excitation–contraction genes differentially regulated in DM versus SF-hCO cardiomyocyte populations from snRNA-seq data are colored in Fig. 2. Created with BioRender.com. Data are the mean ± s.d. Two-tailed Mann–Whitney test (**b**), two-tailed Welch’s *t*-test (**c** and paced data in **f** and **g**), mixed-effects testing with Dunnett’s multiple-comparison tests (**e**) and Kruskal–Wallis test with Dunn’s multiple-comparison tests (**f** and **g**) were performed. ****P < 0.0001. Source data

**Extended Data Fig. 5:**
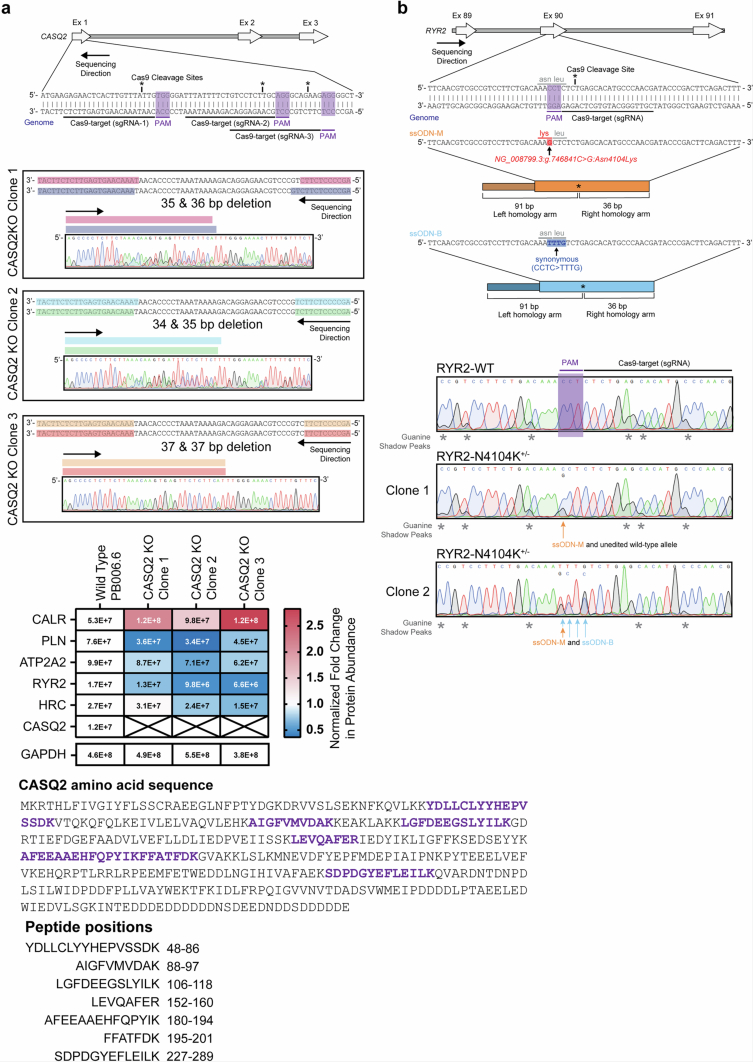
Generation of *CASQ2*^−/−^ and *RYR2*^N4104K/+^cell lines using CRISPR. **a**, Schematic of the CRISPR editing protocol and SANGER sequencing for *CASQ2*^−/−^. This includes proteomics on DM-hCOs confirming knockout of *CASQ2* and other SR related proteins and identified CASQ2 peptides. **b**, Schematic of the CRISPR editing protocol and SANGER sequencing for *RYR2*^N4104K/+^.

One aspect of hPS cell-derived cardiomyocyte immaturity is the high spontaneous contraction rate^37,38^. The decreased rate with AMPK activation in DM-hCOs is consistent with activating mutations in AMPK (gamma 2 subunit) decreasing heart rate in humans^39^. Spontaneous contraction is driven by extracellular membrane currents (*I*_f_) and intracellular sarcoplasmic reticulum calcium release^40,41^. In this series of experiments, we investigated the mechanisms underpinning the reduced rate in DM-hCOs. Both SF-hCOs and DM-hCOs have similar calcium sensitivities for force and no calcium–rate relationship, thus ruling out changes in calcium sensitivity (Fig. 4a). We blocked *I*_*f*_ using 1 μM cilobradine, because the clinically approved compound ivabradine at <10 μM only partially blocks *I*_f_^42^ and prolonged Tr50 at higher concentrations (data not shown). Following blockade of *I*_f_ with 1 μM cilobradine, hCOs became dormant with ‘bursts’ of activity, which were less common in DM-hCOs versus SF-hCOs (Fig. 4b). The ability to block contractile activity in DM-hCOs allowed us to perform post-rest-potentiation experiments, which enable measurement of functional sarcoplasmic reticulum in cardiac muscle^43^. After a 10-s pause, the increase in force of the first re-paced contraction is indicative of increased sarcoplasmic reticulum filling. In DM-hCOs this was 36% (Fig. 4c), which is similar to human hearts (43%) at the same time interval^43^. This increase no longer occurs when sarcoendoplasmic reticulum calcium ATPase (SERCA) is inhibited using 5 μM thapsigargin, thus confirming a functional sarcoplasmic reticulum in DM-hCOs (Fig. 4c). Three-dimensional transmission electron microscopy of a representative section in a DM-hCO also revealed the presence of an extensive sarcoplasmic reticulum network (Fig. 4d), which was confirmed in both SF-hCO and DM-hCO sections (Supplementary Fig. 18). The contribution of sarcoplasmic reticulum calcium release on rate control was assessed by blocking RyR2 with 25 μM ryanodine^44^. This decrease in rate was greater in DM-hCOs (15%) compared to SF-hCOs (6%; Fig. 4e). Force remained constant and the time from 50% activation to peak (Ta50) increased, which remained elevated when paced at 60 bpm to correct for frequency-dependent acceleration of relaxation (Fig. 4f). To further confirm the role of sarcoplasmic reticulum handling in dictating hCO rate, we knocked out the sarcoplasmic reticulum calcium buffering protein *CASQ2* using CRISPR gene editing (Extended Data Fig. 5a). In comparison to an isogenic control, the rate declined in both SF-hCOs and DM-hCOs (Fig. 4g). There were no changes in force and Ta50 was elevated in DM-hCOs when paced at 60 bpm (Fig. 4g). This preserved force combined with increased Ta50 are consistent with sarcoplasmic reticulum blockade in larger mammals and experiments performed with higher extracellular calcium concentrations^41,45,46^ and this is potentially due to L-type calcium channel compensation^47,48^. Collectively, these data confirm that hCO rate is regulated by *I*_f_ and the sarcoplasmic reticulum, and DM-hCOs are more stable under *I*_f_ blockade.

The stability of DM-hCOs under *I*_f_ blockade offered the opportunity to model arrhythmic risk caused by sarcoplasmic reticulum protein mutations. The *CASQ2* knockout and a pathogenic variant *RYR2*^+/N4104K^, which increases risk of arrhythmia in humans^49^, were used (Extended Data Fig. 5b). Using the post-rest-potentiation protocol, there was increased ectopy in *CASQ2*^−/−^ and *RYR2*^+/N4104K^ DM-hCOs (Fig. 4h) indicating increased propensity for arrhythmia.

The decreased rate and increased stability under *I*_f_ blockade in DM-hCOs is underpinned by expression changes in excitation–contraction coupling genes. In DM-hCOs versus SF-hCOs, *PLN* increased, *CACNA1D* decreased (with a concomitant increase in *CACNA1C*) and *GJA1* increased (Extended Data Fig. 2a,c,d and Fig. 4i). Phospholamban (encoded by *PLN*) reduces sarcoplasmic reticulum calcium loading, Cav1.3 (encoded by *CACNA1D*) has been shown to control rate^50^ and CX43 (encoded by *GJA1*) enhances cell–cell coupling altering rate in hPS cell-derived cardiomyocytes^51^. Collectively, these may lead to the reduced rate and stability in DM-hCOs.

### DM-hCOs recapitulate responses to cardioactive drugs

DM-hCOs were benchmarked using 12 comprehensive in vitro pro-arrhythmia assay (CiPA) compounds that bind the human ether-or-go-go channel with different risk stratifications for arrhythmia^52^ and a set of 17 boutique cardioactive compounds with diverse actions.

**Extended Data Fig. 6:**
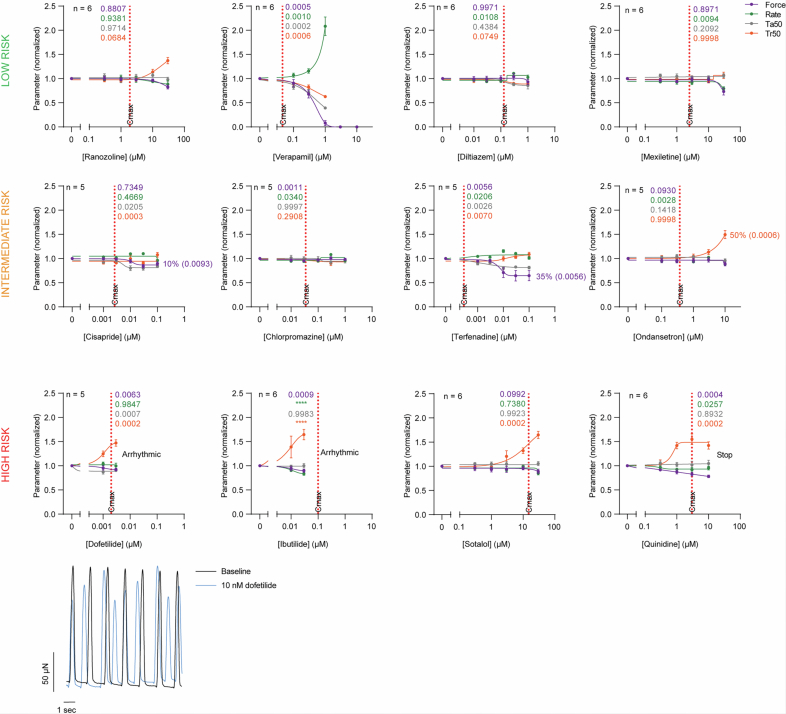
DM-hCOs predict low and high risk CiPA compounds. Functional parameters in response to 12 CiPA compounds. n = hCOs. Data are mean ± standard deviation of functional parameters normalized to non-dosed hCOs then DMSO controls. Richard’s 5 parameter dose-response was used to fit the curves. Mixed-Effects testing with Dunnett’s multiple comparison tests were used to calculate corrected p-values which are only shown for the concentration closest to Cmax. Source data

For the CiPA compounds, Tr50 was the most predictive parameter for risk stratification (Extended Data Fig. 6). All high-risk CiPA compounds that we tested increased Tr50 by 32–64% at the concentration closest to C_max_. Further increases in concentrations either stopped contraction (quinidine) or induced arrhythmias (ibutilide and dofetilide). Low-risk compounds (ranolazine, mexiletine, diltiazem and verapamil) did not increase Tr50, even at supraphysiological concentrations. None of the intermediate-risk compounds (cisapride, terfenadine, ondansetron and chlorpromazine) increased Tr50 at C_max_. Concentration-dependent responses above C_max_ were variable in this group. Cisapride and terfenadine lowered force, and odansetron substantially increased Tr50 (50%). Together, this indicates that the hCOs are reliable in segregating low-risk and high-risk compounds, but assigning intermediate risk is more challenging.

**Extended Data Fig. 7:**
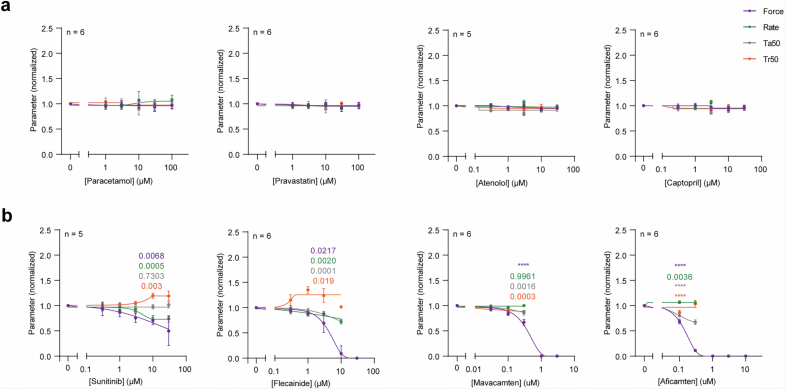
DM-hCOs predict negative inotropes. **a**, DM-hCO responses to benign drugs (paracetamol and pravastatin) or drugs inhibiting the impact of systemic factors (atenolol and captopril). **b**, DM-hCO responses to negative inotropes. n = hCOs. Data are mean ± standard deviation of functional parameters normalized to non-dosed hCOs then DMSO controls. Richard’s 5 parameter dose-response was used to fit the curves. Mixed-Effects testing with Dunnett’s multiple comparison tests were used to calculate corrected p-values which are only shown for the lowest concentration with a decline in force. **** P < 0.0001. Source data

Inert compounds paracetamol and pravastatin did not alter any of the contractile parameters (>10%) at the highest concentrations (Extended Data Fig. 7a). Drugs inhibiting systemic cardiovascular factors including atenolol (β-adrenoreceptor inhibitor) and captopril (angiotensin-converting enzyme inhibitor) also did not alter contractile parameters (>10%; Extended Data Fig. 7a), indicating limited basal adrenergic drive and renin–angiotensin signaling in DM-hCOs.

Negative inotropes sunitinib (RTK inhibitor), verapamil (*I*_Ca,L_ inhibitor), flecainide (*I*_Na_ inhibitor), mavacamten (myosin conformation) and aficamten (myosin conformation) all decreased force in a concentration-dependent manner (Extended Data Fig. 7b). Flecainide also increases Tr50 at low concentrations (as expected). Other contractile parameters are impacted as forces substantially decline, but are no longer as accurate.

**Extended Data Fig. 8:**
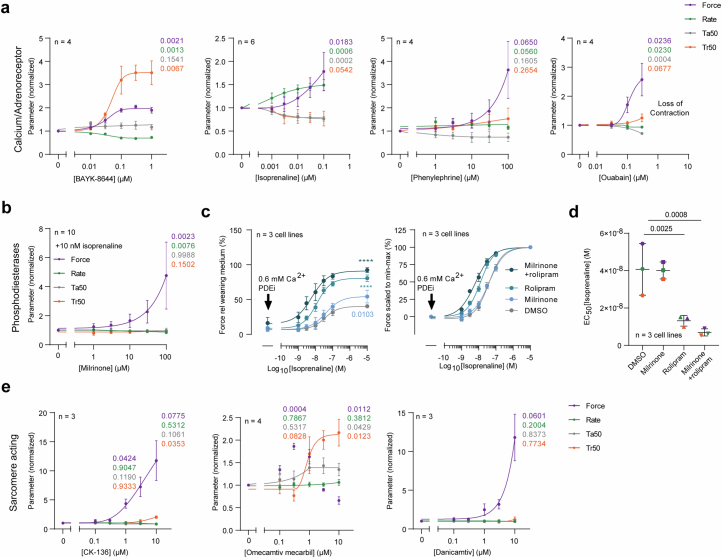
DM-hCOs to predict positive inotropes. **a**, DM-hCO responses to drugs modulating L-type calcium channel (BAYK-8644), β-adrenergic signaling (isoprenaline), α_1-_adrenergic signalling (phenylephrine) or Na+, K+ - ATPase (ouabain). n = hCOs. **b**, DM-hCO responses to phosphodiesterase inhibition using milrinone (PDE3/PDE4) and rolipram (PDE4). n = hCOs. **c**, Isoprenaline dose-response curves with 10 μM and/or 30 μM milrinone. n = experimental averages from 3 different cell lines. **d**, EC_50_ of isoprenaline from the min-max scaled curves in **c**. **e**, DM-hCO responses to sarcomeric acting inotropes targeting troponin (CK-136) or myosin (omecamtiv mecarbil and danicamtiv). All experiments were performed at 0.6 mM Ca^2+^ by mixing weaning medium made in RPMI and DMEM base. Data are mean ± standard deviation. Functional parameters were normalized to non-dosed hCOs then DMSO controls (**a**, **b**, **e**) or as described on the y-axis (**c**). Richard’s 5 parameter dose-response was used to fit the curves. Mixed-Effects testing with Dunnett’s multiple comparison tests (**a**, **b**, **e**), two-way ANOVA with Dunnett’s multiple comparison tests relative to DMSO (**c**) and one-way ANOVA with Dunnett’s multiple comparison tests relative to DMSO (**d**). Corrected p-values are only shown for the highest concentration and intermediate concentrations where Tr50 is impacted (**a**, **b**, **e**). **** P < 0.0001. Source data

Inotropic compounds were tested at 0.6 mM Ca^2+^ given the higher calcium sensitivity in DM-hCOs (Fig. 4a) compared to the human heart (~2.6 mM)^53^. BAYK-8644 (*I*_*Ca*,L_ activator) increased force, but with a large liability on diastolic function with a 250% Tr50 increase. Isoprenaline (β-adrenoreceptor agonist) increased rate in a concentration-dependent manner, and at higher concentrations increase force while decreasing Ta50 and Tr50 (Extended Data Fig. 8a). Phenylephrine (α_1_-adrenorecptor agonist) increased force at the highest concentrations (Extended Data Fig. 8a). Ouabain (*I*_Na/K_ inhibitor) increased force, before contraction cessation at higher concentrations (Extended Data Fig. 8a). Milrinone, a phosphodiesterase 3 (PDE3) inhibitor, increased the force of contraction in the presence of 10 nM isoprenaline, with limited changes in other parameters (<10%; Extended Data Fig. 8b). PDE inhibition, particularly PDE4 by rolipram, increased the magnitude and potency of the isoprenaline response in DM-hCOs from multiple cell lines (Extended Data Fig. 8b).

**Fig. 5:**
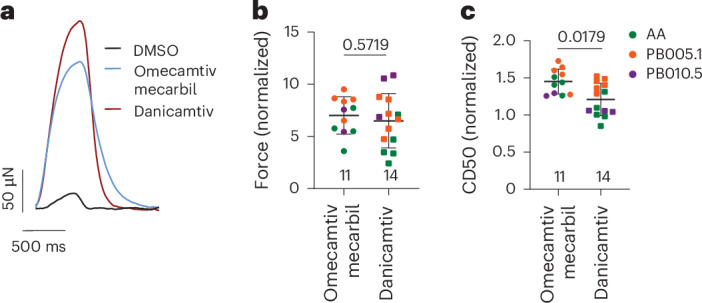
Myosin activators differentially affect contraction duration in DM-hCOs. **a**–**c**, Testing of omecamtiv mecarbil and dancamtiv at C_max_ values, 1 μM and 8 μM, respectively. Experiments were performed at 0.6 mM Ca^2+^ by mixing weaning medium made in RPMI and DMEM base. **a**, Representative force curves. **b**, Force of contraction normalized to pre-drug baseline. *n* = hCOs from three different cell lines (AA, PB005.1 and PB010.5). **c**, Contraction duration between 50% activation and 50% relaxation (CD50). Data are the mean ± s.d. Two-tailed Mann–Whitney tests (**b** and **c**) were performed. Source data

For sarcomeric acting inotropes, CK-136 (nelutroctiv, troponin activator under clinical assessment^54^), increases force with increased Tr50 (102%) at the highest concentration tested (Extended Data Fig. 8c). Omecamtiv mecarbil (myosin activator) increases force in a concentration-dependent manner until >1 μM where it starts to substantially increase Tr50, with force declines at higher concentrations (Extended Data Fig. 8c). Danicamtiv (myosin activator) substantially increases force with limited increases (20%) in Tr50 (Extended Data Fig. 8c). Specifically, when comparing omecamtiv mecarbil and danicamtiv at their higher serum concentrations in the clinic (1 μM (ref. ^55^) and 8 μM (ref. ^56^), respectively), we found that danicamtiv consistently increased force with less contraction duration liability of omecamtiv mecarbil in DM-hCOs from multiple cell lines (Fig. 5a–c).

DM-hCOs accurately predict many drug responses with similar half-maximal inhibitory/effective concentration (IC_50_/EC_50_) values to those reported in the literature^54,56–67^ (Extended Data Table 1). Positive inotropes increased force in our high calcium (1.8 mM) standard culture medium (Supplementary Fig. 19), which is important for screening applications in the future. However, in comparison to adult hearts, DM-hCOs had a higher calcium sensitivity, PDE4 > PDE3 activity (although similar EC_50_ for milrinone as the human heart) and less sensitivity to α_1_-adrenoreceptor stimulation. These features are all consistent with gene expression in DM-hCOs, which was lower for *PDE3* and *ADRA1A* than human heart, and higher for *PDE4* than human heart.

### DM-hCOs model features of DSP cardiomyopathy

**Fig. 6:**
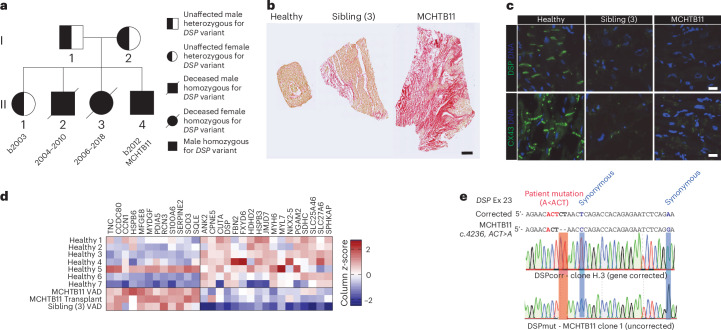
Characterization of DSP cardiomyopathy biopsy samples and generation of CRISPR-corrected hiPS cells. **a**, Family tree. Participant for iPS cell modeling is MCHTB11. **b**, Picrosirius red staining of cardiac biopsy samples from a healthy donor heart or at time of left ventricular assist device implantation for the patients. Scale bar, 500 μm. **c**, Staining of cardiac biopsy samples in **b** for DSP and CX43. Scale bar, 20 μm. **d**, Heat map of DSP signature protein expression in different human heart biopsy samples. **e**, Participant-specific and CRISPR-corrected hiPS cell lines were created using the strategy presented in Supplementary Fig. 21a. Source data

Desmoplakin (DSP) cardiomyopathies are driven by complex cell–cell interactions and functional changes^68^. We identified a participant with dilated cardiomyopathy and arrhythmia (MCHTB11), diagnosed with a homozygous 2-bp deletion in the *DSP* gene (*DSP* c.4246_4247del; p.Leu1416AsnfsTer23). This results in leucine being replaced by asparagine at amino acid position 1416, followed by a termination codon after 22 amino acids in the new reading frame. The variant affects the longer isoform of DSP (NM_004415.3; 2,871 amino acids), but not the shorter isoform (NM_001319034.2; 2,428 amino acids). The family was screened, and the parents were found to be heterozygous, and two deceased siblings were homozygous for the mutation (Fig. 6a). The cardiac pathology was associated with substantial cardiac fibrosis (Fig. 6b) and loss of DSP at the junctions with CX43 dysregulation (Fig. 6c). Proteomic profiling revealed that a diverse array of factors including extracellular matrix, metabolism regulators, ion channel regulators, sarcomeric regulators and growth factors were dysregulated in diseased hearts (Fig. 6d and Supplementary Table 3).

We investigated mice with a similar truncating mutation (*Dsp* c.4315_4318dup; p.Leu1439fs)^69^, yet this model failed to result in any remodeling or fibrosis (Supplementary Fig. 20a–e). This was potentially because there was no loss of DSP at the junctions (Supplementary Fig. 20d). We next created an induced pluripotent cell line (DSPmut) and corrected line (DSPcorr) using simultaneous CRISPR correction with human induced pluripotent stem (hiPS) cell reprogramming^70^ (Fig. 6e and Supplementary Fig. 21a). Using lactate-enriched hPS cell-derived cardiomyocytes cultured in 2D in both SF and DM conditions, we found that DSP was severely reduced and CX43 dysregulated in DSPmut relative to DSPcorr (Supplementary Fig. 21b). There was a clear difference in contraction rate between DSPmut and DSPcorr (Supplementary Fig. 21c), making it challenging to accurately correct for rate or normalize by pacing^71^. As enriched 2D hPS cell-derived cardiomyocytes are also unable to recapitulate the increased fibrosis, we chose to explore the use of the multicellular hCOs as a model.

**Fig. 7:**
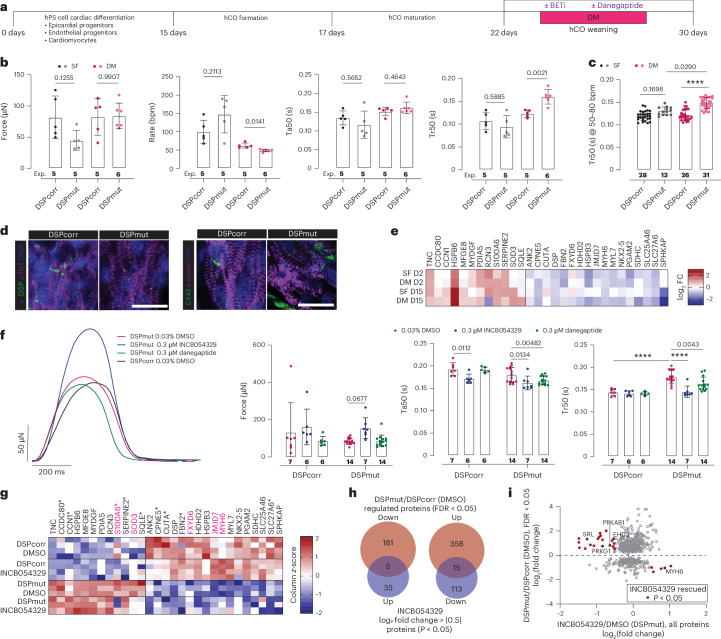
Modeling DSP cardiomyopathy and identification of therapeutic candidates in DM-hCOs. **a**, Schematic of assessment in hCOs. **b**, Contraction parameters of SF-hCOs and DM-hCOs from DSPcorr and DSPmut lines. *n* = experiment means from Extended Data Fig. 9a. **c**, Tr50 in hCOs beating between 50 and 80 bpm (Extended Data Fig. 9b). *n* = hCOs pooled from all experiments in Extended Data Fig. 9a. **d**, Staining of DM-hCOs for DSP and CX43. Scale bars, 20 μm. Representative of *n* = 3–4 biological replicates. **e**, Heat map of DSP signature protein fold change in immature 2-day and mature 15-day SF-hCOs and DM-hCOs in DSPmut versus DSPcorr lines. *n* = 2–3 experiments per group, each with 3 pooled hCOs per sample. Outliers with undetected or very low protein expression were removed from the analysis. **f**, Representative force traces of 60-bpm paced DM-hCOs including analysis of force and kinetic parameters with INCB054329 or danegaptide treatment. *n* = hCOs pooled from 3–4 experiments. Baseline functional parameters are in Extended Data Fig. 9c. **g**, Heat map of DSP signature proteins in DM-hCOs in DSPmut versus DSPcorr lines with and without INCB054329. *n* = 3–4 experiments, each with 3 pooled hCOs per sample. All FDR < 0.05 except those with *P < 0.05 in DSPmut versus DSPcorr DM-hCOs. Proteins reverted by INCB054329 are highlighted in magenta. **h**, Venn diagrams of DSPmut versus DSPcorr (FDR < 0.05 or *P* < 0.05) that are also differentially regulated by INCB054329 in DSPmut (*P* < 0.05). **i**, Plot highlighting the differentially regulated proteins in DSPmut versus DSPcorr (FDR < 0.05) that are also differentially regulated by INCB054329 in DSPmut (*P* < 0.05). Data are the mean ± s.d. Brown–Forsythe and Welch with Dunnett’s T3 multiple-comparison tests comparing DSPmut to DSPcorr (**b**), Kruskal–Wallis with Dunn’s multiple-comparison tests (**c**) and two-way analysis of variance with Sidak’s multiple-comparison test between DSPcorr and DSPmut for DMSO or relative to DMSO for DSPmut (**f**) were performed. ****P < 0.0001. Source data

**Extended Data Fig. 9:**
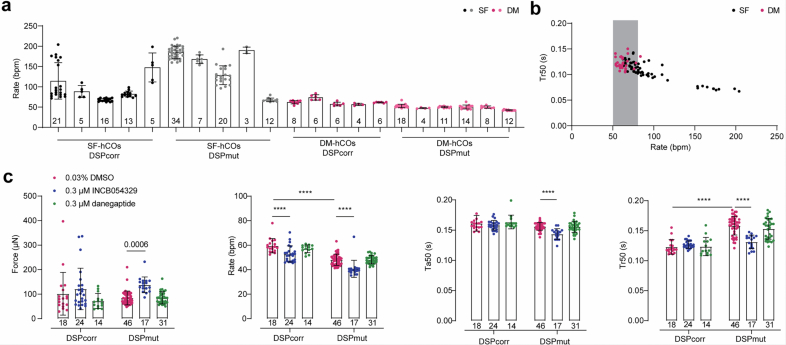
Modelling and drug testing in DSPmut hCOs. **a**, Rate in hCOs in different experimental batches. n = hCOs and each bar is a different experiment. **b**, Tr50 versus rate in DSPcorr hCOs with 50–80 bpm highlighted. n = 90 hCOs from all DSPcorr experiments in **a**. **c**, Contraction parameters with treatment of INCB054329 and danegaptide in DM-hCOs (as per Fig. 7a). n = hCOs pooled from 3–4 experiments. Two-way ANOVA with Sidak’s multiple comparison tests between lines for DMSO or relative to DMSO for DSPmut (**c**). **** p < 0.0001. Source data

Both SF-hCOs and DM-hCOs were created from DSPcorr and DSPmut lines (Fig. 7a). DM-hCOs had a consistent contraction rate, which revealed a 30% increase in Tr50 between DSPmut versus DSPcorr (Fig. 7b), indicative of stiffening and diastolic dysfunction. To ensure that this phenotype was not being masked by the variable contraction rate of SF-hCOs (Extended Data Fig. 9a), the Tr50 was compared in hCOs within a range of 50–80 bpm where Tr50 is not rate dependent (Extended Data Fig. 9b). Within this subset, Tr50 remained unaltered in SF-hCOs, while Tr50 in the DSPmut DM-hCOs was significantly elevated (Fig. 7c). This is consistent with human data where echocardiography revealed mitral valve ratio of peak early to late diastolic filling velocities (E′/A′) was elevated at 1.8 (2.3 in sibling 3), indicative of diastolic dysfunction.

DSPcorr DM-hCOs displayed normal junctional expression of DSP and CX43, but largely absent DSP expression and aberrant CX43 localization in the DSPmut line, which also had disordered sarcomeres (Fig. 7d). Induction of fibrosis marked by tenascin C was also confirmed in both SF-hCOs and DM-hCOs (Supplementary Fig. 22). Proteomics revealed that the perturbed markers (Fig. 6d) were most consistently differentially regulated in DM-hCOs after 15 days in comparison to SF-hCOs or hCOs at an initial 2-day time point (Fig. 7e). Key examples include CCDC80, which is a cardiac fibroblast gene that may play a role in heart failure^72^, and FXYD6 which regulates the sodium–potassium ATPase^73^.

To improve the relaxation phenotype, we trialed two drugs: INCB054329 and danegaptide. INCB054329 is a bromodomain and extra-terminal protein inhibitor that we recently discovered can correct inflammation-induced diastolic dysfunction^74^. Danegaptide is a gap junction enhancer^75^ that was also assessed due to the CX43 remodeling observed (Fig. 7d), as well as recent interest in CX43 gene therapies for arrhythmogenic cardiomyopathy^76^. INCB054329 and danegaptide had no substantial effects in the DSPcorr DM-hCOs other than a slightly reduced rate for INCB054329 (Extended Data Fig. 9). In DSPmut DM-hCOs, INCB054329 increased force, slightly decreased rate and fully corrected the Tr50 phenotype (Extended Data Fig. 9c). Under 60-bpm paced conditions, danegaptide slightly improved Tr50, while INCB054329 fully reverted the phenotype in DSPmut DM-hCOs (Fig. 7f).

To determine the underlying mechanisms of how INCB054329 confers improved function, we performed proteomics. There was extensive regulation of the proteome with 525 differentially regulated proteins (false discovery rate (FDR) < 0.05) in DSPmut versus DSPcorr DM-hCOs (Supplementary Fig. 23a,b) resulting in a clear shift when plotted using principal components 1 and 2 (Supplementary Fig. 23c), while INCB054329 differentially regulated a smaller set of proteins (Supplementary Fig. 23d). A key signature is extracellular matrix and immune regulators that were increased in both DSPmut DM-hCOs and DSPmut human samples (Supplementary Fig. 24a,b). We expanded our analysis and identified ‘DSP signature proteins’ as those statistically differentially regulated in this dataset and changed in expression log_2_ > |0.5| in DSPmut human hearts (Fig. 6d) and the independent DSPmut DM-hCO dataset (Figs. 6d and 7e,g). Of the identified DSP signature proteins, INCB054329 reverted a small subset of the DSP signature proteins (Fig. 7g–i). With INCB054329 treatment, DSP remained decreased and the upregulated extracellular matrix associated proteins TNC, CCDC80, CCN1, MFGE8, RCN3 and SERPINE2 remained elevated (Fig. 7g). Instead, S100A6 and SOD3 were reverted via decreased expression, and FXYD6, JMJD7 and MYH6 were reverted via increased expression (Fig. 7g). Major changes in MYH6 expression are worth noting as there is a 28–166-fold decrease in the DSPmut human heart biopsy samples.

## Discussion

In this study, we use our Heart-Dyno hCO platform to investigate whether key in vivo signatures we identified in human hearts play a role during in vitro cardiac maturation^17^. Together, MK8722 and DY131 were the most effective drivers of increased maturation. In contrast to previous studies^77^, activation of ERR (using DY131) had a relatively subtle impact, whereas activation of AMPK using MK8722 dramatically enhanced maturation. These DM stimuli induced a uniform response across the hCOs (Fig. 3i,j). Typically diffusion barriers limit effective oxygen concentrations in larger tissue engineered formats, which restricts the size of thick cardiac muscle to ~150 μm before oxygen becomes limiting and alters metabolism^78^. hCOs are ~70–100-μm thick and, therefore, do not have a restrictive diffusion barrier, which is why there is not a graded response across the hCOs. Critically, DM was most effective following—but not during—the phase where hCOs are metabolically switched to fatty acid oxidation^4^. This may be because during this phase the cardiomyocytes are already near maximum capacity as indicated by our data profiling oxidation rate over the full hCO culture time course (Fig. 3g). Therefore, additional metabolic stress under conditions of low glucose provision and high fatty acid provision may be detrimental. These data further support our previous findings that oxidative metabolism is not just a feature, but a driver of cardiac maturation^4,79^.

The acute application of DY131 and MK8722 induced a similar signature to pacing with over 48% of activated phosphopeptides shared, including an AMPK signaling network that we identify to underpin maturation in pacing and DM-hCO protocols. High endogenous contraction rates alone do not promote maturation, as PB010.5 SF-hCOs had endogenous beating rates of >150 bpm (Fig. 1f), but low levels of cTnI expression that were greatly enhanced in DM-hCOs (Fig. 1g). Therefore, increases in metabolic capacity via stimuli-driven processes are essential for maturation. Even after the DY131 and MK8722 are removed, the increase in maturation markers such as cTnI and reduced endogenous contraction rate are sustained (Fig. 1b,c) and this is consistent with DM-hCOs maintaining a stable maturation cellular state. The increased metabolic capacity may be a key adaptation during postnatal maturation to enable large increases in demand during exercise or stress. It will therefore be interesting to determine whether exercise is required to maintain the maturation program in vivo.

One key feature of DM-hCOs is a more consistent rate across different hPS cell lines. This made it possible to (1) assess sarcoplasmic reticulum function under reduced *I*_f_ in >90% of DM-hCOs, and (2) assess ectopy as a surrogate readout of arrhythmia when altering sarcoplasmic reticulum-related proteins. Automaticity in hPS cell-derived cardiomyocytes results from relatively high expression of *HCN4*, *CACNA1H* and *SLC8A1*, with low expression of *KCNJ2* (ref. ^38^). Alternative mechanisms may be responsible for reduced rates in our DM-hCOs including a switch from *CACNA1D* to *CACNA1C*, and increased CX43 expression as key changes consistent with human heart maturation (Fig. 4).

The *DSP* mutation induced dysregulation of proteins across a broad range of biological processes in human heart tissue (Fig. 6d) and DM-hCOs (Fig. 7e,g) and may require multicellular populations to model effectively. DM-hCOs do not display irregular contractions and are likely modeling the initial stages of disease, which can take years to result in systolic decline and episodes of ventricular arrhythmia^80^. In the future it will be interesting to determine whether diastolic dysfunction precedes or progressively develops with systolic dysfunction in patients with *DSP* mutations. In addition, as our hCOs have left ventricular identity and *DSP* mutations predominantly impact the left ventricle^80^, it may be critical to have a model of the left ventricular myocardium to recapitulate the pathology. Treatment with the bromodomain and extra-terminal protein inhibitor INCB054329 rescued the relaxation defect in DSPmut DM-hCOs without impacting the abundance of the key fibrotic regulators. Instead, MYH6 expression was restored (Fig. 7g). Mutations in the fast isoform of myosin heavy chain (MYH6) drive a diverse range in human cardiac pathologies including structural remodeling and dilated cardiomyopathy^81^. This occurs even though the MYH6 protein abundance is only ~7% of the myosin heavy chain protein in humans with the slow isoform of myosin heavy chain (MYH7) being the predominant form^81^. In patients with idiopathic dilated cardiomyopathy, MYH6 is decreased, and is increased again in patients with improved cardiac function in response to beta-blocker therapy^81^. Therefore, bromodomain and extra-terminal protein inhibitors may be beneficial for DSP cardiomyopathy and prevent myosin heavy chain isoform switching.

## Methods

### Human heart tissue and hPS cell lines

Ethical approval for the generation and/or use of human heart tissue and hPS cells was obtained from QIMR Berghofer’s Ethics Committee and Murdoch Children’s Research Institute (MCRI) Ethics Committees and were carried out in accordance with the National Health and Medical Research Council of Australia regulations. Informed consent was obtained from all participants for collection and storage of human material (blood and tissues) and the derivation and use of iPS cells. hPS cells were also obtained from WiCell, Coriell and the CIRM hPS cell repository funded by the California Institute of Regenerative Medicine (Supplementary Table 4). hPS cell lines are available upon request with appropriate agreements and ethical approvals.

hPS cell lines were maintained in mTeSR Plus (StemCell Technologies) in Matrigel-coated flasks (Millipore) and passaged using ReLeSR (StemCell Technologies). Karyotypes were routinely checked by G-banding (Sullivan Nicolaides) and molecular karyotyping analysis (Victorian Clinical Genetics Service and Ramaciotti Centre for Genomics). All cultures were routinely tested for mycoplasma. HES3 and all derived clones had a duplication at 20q20.11.

### DSP mouse experiments

Animal experiments were conducted in accordance with the relevant codes of practice for the care and use of animals for scientific purposes as stipulated by the National Health and Medical Research Council of Australia and conducted with approval from the Animal Ethics Committee at the MCRI (A958). DSPrul (RB156Bnr/Ei-*Dsp*^*rul*^/GrsrJ) mice^69^ were obtained from The Jackson Laboratory (strain no. 005362). All mice were maintained in a 12–12-h light–dark cycle at a temperature of 22 °C ± 1 °C and humidity of 40–70%, and provided ad libitum access to food and water during the study duration. At 45 weeks of age, mice were placed under inhalant anesthesia followed by cervical dislocation. Hearts were excised, weighed and fixed in 4% paraformaldehyde in PBS for 24 h, 30% sucrose diluted in PBS for 24 h at 4 °C and embedded in OCT for cryosectioning. Heart tissue used for transmission electron microscopy was taken from the left ventricle (1 mm^3^pieces) and placed in 1% glutaraldehyde, 1.5% paraformaldehyde and 0.1 M cacodylate diluted in PBS. Heart weights were normalized to tibia length as indicated. Heart structure was assessed by H&E staining, and fibrosis was assessed histologically using Masson’s Trichrome staining on sections of left ventricle cut to 8 μm. DSP and cardiac troponin T expression and localization in cardiomyocytes were assessed using immunofluorescence labeling.

### Generation of hPS cell transgenic lines

For the LED pacing line, neomycin resistance followed by CAG-C1V1(E122T/E162T) channel rhopdopsin-P2A-super ecliptic pHurlion was cloned into an AAVS1 targeting construct with ~800-bp homology arms. For the TCF21 reporter line, a homologous directed recombination template was synthesized as depicted in the relevant figure.

Reprogramming and gene-editing factors (Cas9-Gem mRNA, TCF21:CreERT2 template and plasmid encoding TCF21-specific single guide RNA (sgRNA)) were introduced into peripheral blood mononuclear cells using the Neon transfection system (Thermo Fisher Scientific, 1150 V, 30 ms, two pulses). Transfected cells were plated over three wells of a Matrigel/mouse embryonic fibroblast-coated six-well dish in StemSpan Media. E8 medium was added to each well 2 days after transfection and half medium changes were performed every other day until adherent iPS cell colonies became visible (~1 week after transfection) when full E8 media changes were performed. Individual iPS cell colonies were isolated and expanded in E8 medium.

Homozygous knock-in of the CreERT2 was confirmed by PCR analysis using primers flanking the target site. A clone harboring homozygous insertion of CreERT2 was selected for incorporation of the fate-mapper cassette into the *EEF2* locus. Gene-editing factors (Cas9-Gem mRNA, EEF2 fate-mapper HDR template and plasmid encoding EEF2-specific sgRNA) were introduced into iPS cells using the Neon transfection system (1,100 V, 30 ms, one pulse). Transfected cells were plated over four wells of a Matrigel-coated six-well dish in mTesR medium supplemented with 10 μM Y-27632 (StemCell Technologies) and removed from medium after 24 h. Successfully edited iPS cell colonies (expressing EYFP) were identified by fluorescence microscopy. EYFP^+^ colonies were isolated and expanded in E8 medium. A clone with a high proportion of EYFP^+^ cells was selected for subcloning to attain a pure population. Subcloning was performed by dissociating cells with TryPLE and plating at low density in mTesR supplemented with Y-27632 (removed from medium after 24 h). Individual colonies were picked and expanded in E8 medium. One of these clones was confirmed by flow cytometry to consist entirely of EYFP^+^ cells and showed homozygous knock-in of the EEF2 fate-mapper transgene as evidenced by PCR analysis using primers flanking the *EEF2* target site. sgRNA details are provided in the relevant figures.

For the generation of DSP mutant and corrected iPS cell lines, episomal reprogramming plasmids and gene-editing factors were introduced into participant peripheral blood mononuclear cells (MCRI Biobank ID; MCHTB11.9) as above. Transfected cells were plated over three wells of a Matrigel-coated six-well dish and then cultured in Essential 8 medium. Media changes were performed every other day. Individual iPS cell colonies were picked and expanded in Essential 8 medium. Individual iPS cell colonies were genotyped by PCR using primers (AGAAAACGCCCTTCAGCAA and CTCCAGCTTCTTCCTCTTGC) flanking the participant-specific mutation. Amplicons were sequenced via Sanger protocols to confirm gene correction and absence of indel mutations.

For the generation of the *CASQ2*^−/−^ and *RYR2*^+/N4104K^ hPS cell lines, 2 h before electroporation, medium on the pluripotent stem cells was changed to fresh mTeSR Plus supplemented with 10 µM ROCK inhibitor Y-27632. For the *CASQ2*^−/−^ mutation, sgRNAs (Synthego) were conjugated to TrueCut Cas9 Protein v2 (Thermo Fisher Scientific) in a 9:1 molar ratio of sgRNA:Cas9 in a 10 µl reaction. For the *RYR2*^+/N4104K^, mutation 1 µl of 60 µM Alt-R CRISPR–Cas9 sgRNA (Integrated DNA Technologies) was conjugated to 1 μl of 3 mg ml^−1^ TrueCut Cas9 Protein v2, in a 10 µl reaction. Cells were harvested with ReLeSR and 1.6 × 10^5^ cells resuspended in buffer R (12 μl) with the sgRNA–Cas9 ribonucleoprotein complex prepared above. For *RYR2*^+/N4104K^ mutation, 0.6 μl of each 100 μM single-stranded oligodeoxynucleotide was added. sgRNAs and single-stranded oligodeoxynucleotide details are provided in the relevant figures and Supplementary Tables 5 and 6.

hPS cells were transfected using the Neon transfection system 10 μl kit (1200 V, 30 ms, one pulse) and immediately plated onto 24-well tissue culture plates coated with Matrigel, containing mTeSR Plus with 10 µM Y-27632, or 1× CloneR2 (StemCell Technologies). Media were changed to mTeSR Plus the next day without further supplements. Media were changed every 2 to 3 days with mTeSR Plus until hPS cells reached 70% confluency and they were then cryopreserved using mFreSR (StemCell Technologies). Concurrently, hPS cells were harvested for DNA with the DNeasy Blood & Tissue Kit (Qiagen) according to the manufacturer’s instructions.

Amplicons of target sites were generated from DNA of electroporated hPS cells with Hifi Platinum Taq DNA Polymerase High Fidelity (Thermo Fisher Scientific) according to the manufacturer’s instructions. PCR purification was performed with the QIAquick PCR Purification Kit (Qiagen) according to the manufacturer’s instructions. Sanger sequencing was performed by the QIMR Berghofer Analytical Facility using primers in Supplementary Table 7. The sequence traces from the wild-type parental cell lines and the electroporated hPS cell populations were analyzed with Synthego’s ICE Analysis tool (available at https://ice.synthego.com/) to determine editing efficiency.

hPS cell pools with greater than 1% editing efficiency were thawed into T25 flasks for single-cell cloning. Cloning medium was prepared by supplementing mTeSR Plus with 10% CloneR or CloneR2. Cells were passaged using ReLeSR, passed through a 100-μm cell strainer and sorted using a BD FACSAria IIIu Cell Sorter (BD Biosciences) into 96-well plates (one cell per well). FSC/SSC gates were set to identify individual cells and exclude cell debris and clusters of two or more cells. Culture continued in cloning medium for 2–5 days, and once all colonies were over 25% confluency they were each passaged into one well of a 96-well plate and 24-well plate and maintained in mTeSR Plus. The Extract-N-Amp for Blood Kit (Sigma-Aldrich) was used according to the manufacturer’s instructions to extract DNA from the 96-well plate and for PCR amplification of the edited sites. PCR amplicons were purified with the QIAquick PCR Purification Kit and submitted for Sanger sequencing as above. Sanger chromatograms were manually interrogated to determine successful edits. Positive clones were expanded and cryopreserved. hPS cell lines were karyotyped as described above.

### Cardiac differentiation

The single-step ventricular cardiomyocyte and stromal cell differentiation protocol and dissociation and hCO formation was performed as recently described in detail^8,21^. Four days before initiation of differentiation, hPS cells were seeded onto Matrigel-coated T25 tissue culture flasks at a density of 0.6–1.6 × 10^4^ cells per cm^2^ and cultured with mTeSR Plus. Exact plating densities were adjusted for each cell line to achieve a confluency of 50–60% after 3 days. The medium was changed to mTeSR1 for one day. Mesoderm induction was initiated by changing the medium to RPMI 1640 GlutaMAX medium (Thermo Fisher Scientific) supplemented with 2% B27 minus insulin (Thermo Fisher Scientific), 200 µM l-ascorbic acid 2-phosphate sesquimagnesium salt hydrate (l-AA2P; Sigma), 1% penicillin–streptomycin (Thermo Fisher Scientific), 1 µM CHIR99021 (StemCell Technologies), 9 ng ml^−1^ activin A (Thermo Fisher Scientific), 5 ng ml^−1^ BMP4 (Thermo Fisher Scientific) and 5 ng ml^−1^ FGF-2 (RnD Systems) for 3 days with daily media changes. On day 3, the medium was changed to RPMI 1640 GlutaMAX medium supplemented with 2% B27 minus insulin, 200 µM l-AA2P, 1% penicillin–streptomycin and 5 µM IWP4 (StemCell Technologies). From day 6 to 13, differentiating cardiac cells were cultured in the previous medium except B27 minus insulin was exchanged for B27 (with insulin), with media changes every two to three days. On day 13, the medium was refreshed and IWP4 was removed. On day 15, differentiated cardiac cells were digested using 0.2% collagenase type I (Sigma) in 20% fetal bovine serum (FBS; Thermo Fisher Scientific) and PBS (with Ca^2+^ and Mg^2+^) for 60 min at 37 °C in a humidified incubator. Cardiac cells were collected, washed and centrifuged in PBS (without Ca^2+^ and Mg^2+^), then resuspended in 0.25% trypsin-EDTA for 10 min at 37 °C with gentle agitation every minute. Equivolume MEMα GlutaMAX (Gibco) supplemented with 10% FBS, 200 μM l-AA2P and 1% penicillin–streptomycin was added to the hPS cell-derived cardiac cell (hPS cell-CC) suspension, and the mixture was filtered with a 100-μm cell strainer (BD Biosciences).

For pacemaker differentiation, hPS cells underwent the 3-day mesoderm induction as above for ventricular differentiation of hPS cell-CCs. On day 4, 5 μM IWP4, 2.5 ng ml^−1^ BMP4, 5.4 mM SB431542 (Sigma), 0.24 mM all-trans retinoic acid (StemCell Technologies) and 0.5 mM PD173074 (Tocris) were added to RPMI base medium to pattern the cardiac cells. B27 (plus insulin) RPMI basal medium supplemented with 5 μM IWP4 was added for the next 7 days with media changes every 2 to 3 days. On day 15 of differentiation, B27 (insulin minus) DMEM no glucose, no glutamine, no phenol red (Thermo Fisher Scientific) supplemented with 5 mM lactic acid (Sigma) was added for the next 7 days with media changes every 2 to 3 days. On day 22 of differentiation, cells were dissociated as above for ventricular differentiation of hPS cell-CCs.

### hCO formation and culture

Heart-Dyno culture inserts were manufactured with polydimethylsiloxane (PDMS), SYLGARD 184 Silicone Elastomer (CBC Australia) at a 10:1 ratio of base to curing agent. The base and curing agent were thoroughly mixed and degassed in a vacuum chamber. Heart-Dyno plates were cast in a machined aluminum mold (ANFF-SA). After pouring into a mold, the PDMS was degassed again in a vacuum chamber then cured for 1 h at 70 °C.

Heart-Dyno culture plates were fabricated using two different methods. For one method, individual well inserts were manually ‘glued’ into 96-well flat-bottom tissue culture plates (Greiner and Corning) with uncured PDMS (10:1 ratio of base to curing agent) and were left for at least 24 h at room temperature to cure. For the new method, the whole 96-well Heart-Dyno layer was bonded to 96-well flat-bottom tissue culture plates (Greiner and Corning) using chemical bonding and uncured PDMS (10:1 ratio of base to curing agent)^82^.

Tissue culture plates with Heart-Dyno inserts were sterilized in a biosafety cabinet by immersion in 80% vol/vol ethanol for 2 h followed by 1 h of ultraviolet exposure in the biosafety cabinet. Before seeding of hCOs, the HeartDynos were coated with 1–3% wt/vol bovine serum albumin (Sigma; in PBS without Ca^2+^ and Mg^2+^) for 2 h to limit cell adhesion to the PDMS. This was aspirated immediately before seeding.

Before seeding of hCOs, hPS cell-CCs (differentiation described above) were centrifuged at 300*g* for 3 min and resuspended in serum-free medium: MEMα GlutaMAX supplemented with 200 μM l-AA2P, 1% penicillin–streptomycin, 4% vol/vol B27 with insulin, 10 ng ml^−1^ FGF-2 and 10 ng ml^−1^ PDGF-BB (RnD Systems). Each Heart-Dyno plate was seeded with a 3.5 μl mix of 5 × 10^4^ cardiac cells in serum-free medium, 2.6 mg ml^−1^ acid-solubilized collagen I (Devro) that was salt balanced with 10× DMEM (Thermo Fisher Scientific) and pH neutralized with 0.1 M NaOH and 9% vol/vol Matrigel. All components were mixed over ice, as was the manual pipetting of the mix into the Heart-Dyno plates, to prevent premature gelling. After seeding, Heart-Dyno plates were incubated at 37 °C in a 5% CO_2_ humidified incubator for 30–45 min to allow the mix to gel. After gelling, 150 μl of serum-free medium warmed to 37 °C was added to each well. hPS cell-CCs were allowed to condense and self-organize into hCOs for 2 days in the serum-free medium. On day 17, the medium was changed to maturation medium: DMEM, no glucose, no glutamine, no phenol red (Thermo Fisher Scientific) supplemented with 1× GlutaMAX (Thermo Fisher Scientific), 200 μM l-AA2P, 1% penicillin–streptomycin, 4% vol/vol B27 without insulin (Thermo Fisher Scientific), 10 ng ml^−1^ FGF-2, 10 ng ml^−1^ PDGF-BB, 33 μg ml^−1^ aprotinin (Sigma or MedChemExpress), 100 μM palmitate (conjugated to bovine serum albumin in B27, Sigma) and 1 mM glucose (Sigma). Maturation medium was refreshed on day 20. On day 22, the medium was changed to weaning medium. The composition of weaning medium has the following differences to maturation medium: no FGF-2, no PDGF-BB, 10 μM palmitate instead of 100 μM, 5.5 mM glucose instead of 1 mM, and 1 nM recombinant human insulin (Gibco). Weaning medium was refreshed on days 24 and 27. For directed maturation conditions, 2 μM CHIR99021 was added for the formation phase in the first 2 days for hCO formation and the media changes on days 24 and 27 were instead done with weaning medium supplemented with 3 μM DY131 and 10 μM MK8722 (both MedChemExpress). All further media changes were done with weaning medium every 2 to 3 days with no further supplements. For some experiments, VIAFLO 96 (Integra) semiautomated pipetting electronic pipetting systems were used for seeding the hCOs and media changes.

### Chronic pacing conditions

hCOs were chronically paced using different methods. One method used isoprenaline (Sigma; Supplementary Fig. 1). Another used exposure of red-shifted channel-rhodopsin expressing hCOs to pulses of green light using a 96-well green LED array (Lumidox) connected to a Panlab/Harvard Apparatus Digital Stimulator^22^ (Supplementary Fig. 3). For pacing in the minutes range, the Heart-Dyno inserts were also fabricated in a custom plate format to fit into a 24-well plate for acute pacing using a C-Pace Cell Culture EP Stimulator (Ionoptix) using 10 V with 1-ms pulses at 120 bpm (Fig. 1).

### Maturation factors

Various factors were added at the times and concentrations outlined in the different figures. Small molecules GSK4716, DY131, MK8722 and O304 were purchased from MedChemExpress. Interferons IFNγ, IFNλ1, IFNλ2, IFNβ and IFNω were purchased from PeproTech. Fatty acids palmitic acid, oleic acid, myristic acid and linoleic acid were purchased from Sigma.

### 2D culture

Cardiac cell differentiation was followed as per the hCO culture. For 2D experiments in Supplementary Fig. 10, the cells were plated onto gelatin-coated plates. For 2D experiments in Supplementary Fig. 21, the following protocol was used. To enrich for hPS cell-CMs, for the last 5 days of the differentiation, the RPMI 1640 base was exchanged for RPMI 1640, no glucose, and 4 mM lactate was added to the media. Cells were then plated onto laminin-521-coated plates (5 μg ml^−1^) overnight at 4 °C.

2D cells were cultured in CTRL^4^ medium for the first 2 days after plating, after which they were changed to maturation medium^8,21^ without FGF-2 and PDGF-BB. On day 7 (afer seeding), they were changed to weaning medium^8,21^. On day 9, DM conditions received the DY131 and MK8722 compounds for 4 days before a final 2 days of culture in weaning medium.

For functional analysis of 2D cultures, cells were imaged at 50 Hz for 10 s with bright-field microscopy using a Leica Thunder microscope (with LAS X software current version 5.3.0) with a long-working distance ×20 objective. Each well was imaged twice at different locations with high hPS cell-CM coverage. Images were exported as TIF stacks. All TIF stacks were analyzed with MUSCLEMOTION^83^. Every trace generated was manually inspected for correct tracking.

### Force analysis

hCOs were imaged under environmentally controlled conditions at 37 °C with 5% CO_2_ on a Leica Thunder microscope (with LAS X software current version 5.3.0). Image series were taken of each hCO at 50 Hz for 10 s, but up to 40 s for experiments using cilobradine. Contraction analysis was performed using MATLAB scripts available from ref. ^21^ or Tempo.ai analysis software (Dynomics).

For calcium sensitivity experiments, Tyrode’s solution (120 mM NaCl, 5 mM KCl, 22.6 mM NaHCO_3_, 2 mM MgCl_2_ and pH adjusted to 7.4) was used and calcium concentration adjusted using a 0.2 M CaCl_2_ stock solution.

To assess post-rest potentiation, hCOs were pretreated with 1 µM cilobradine (and in some cases 5 µM thapsigargin) for 2 h before the experiment. Pacing of hCOs with field stimulation was performed with custom-designed gold-plated electrode lids at 30–40 mA with 5-ms square pulses for at least 30 s before recordings.

### qPCR

hCOs were manually homogenized in 500 µl TRIzol (Thermo Fisher Scientific) with a stainless-steel ball bearing and 10-s vortex pulses until solubilized. Samples were stored at −80 °C before RNA was extracted using TRIzol per the manufacturer’s instructions. RNA was treated with DNase per the manufacturer’s instructions (Roche) before cDNA synthesis (Thermo Fisher Scientific). Powerup SYBR Green Master Mix (Thermo Fisher Scientific) was used for RT–qPCR using StepOne software v2.3 to determine gene expression (primers are in Supplementary Table 8).

### Immunostaining

hCOs were fixed with 1% paraformaldehyde (Sigma) for 60 min. Cells were stained with primary antibodies (Supplementary Table 9) in 5% FBS (Thermo Fisher Scientific) and 0.2% Triton X-100 (Sigma) in PBS (blocking buffer) at 4 °C overnight on a rocker. Cells were washed 2× for 1 h with blocking buffer and labeled with secondary antibodies (Supplementary Table 9) and Hoechst 33342 at 4 °C overnight on a rocker. Cells were again washed with blocking buffer 2× for 1 h and imaged in the Heart-Dyno or mounted on microscope slides in ProLong Glass (Thermo Fisher Scientific). 2D cells were stained and imaged in a similar manner, but with shorter incubation (~1–2 h) and wash (~5 min) times. Low-magnification images were taken on a Leica Thunder microscope (LAS X software current version 5.3.0). High-magnification images were taken using a Zeiss 780-NLO point scanning confocal microscope (ZEN software Version 3.7) for analysis on a Leica Stellaris 5 (LAS X software current version 5.3.0).

### Drug testing

Individual compounds were purchased from Sigma to form our boutique drug libraries. These were added to hCOs for at least 15 min for equilibration before video recordings. For concentration–response curves, cumulative drug additions were applied to the same hCO.

### Transmission electron microscopy

hCOs were fixed in 2.5% glutaraldehyde in PBS for 1 h at room temperature and then processed for embedding in situ as described previously^84^. For electron tomography, 200–300-nm sections were prepared parallel to the base of the plate on a Leica Ultracut 6 ultramicrotome. The grid was then coated with a thin carbon layer. Tomography was performed as described previously^85^ on a Tecnai F30 transmission electron microscope (FEI) at 300 kV. A dual‐axis tilt series spanning ± 60° with 1° increments was acquired with a Gatan OneView camera under the control of SerialEM software. Tilt series were reconstructed using IMOD (https://bio3d.colorado.edu/imod/) with segmentation performed by density thresholding using the Isosurface Render program in IMOD.

### DSP hCO proteomics

Each sample contained three hCOs and was stored (−20 °C) before preparation. Samples were thawed on ice, and residual supernatant was removed before addition of 150 μl chilled lysis buffer (4% sodium deoxycholate in TRIS-buffered saline). Samples were immediately boiled at 95 °C for 5 min to inactivate endogenous enzymatic activity and assist lysis. Four volumes of chilled acetone were added to each sample, and sonicated in 1-min bouts alternating with incubation on ice for a total of five cycles. Protein was pelleted by centrifugation at 20,000*g* for 30 min at 4 °C. Supernatant was removed, and the protein pellet was washed twice in acetone before resuspending in 150 μl 50 mM triethylammonium bicarbonate buffer (Sigma). Reduction alkylation buffer was added to a final concentration of 10 mM tris(2-carboxyethyl)phosphine (Sigma) and 40 mM 2-chloroacetamide (Sigma, equilibrated with KOH to pH 8) and incubated for 10 min at 45 °C with 1,500 rpm agitation. Next, 3 µg trypsin enzyme mix (Thermo Fisher Scientific) was added to each sample and samples were incubated overnight (17 h) at 37 °C, with 1,500 rpm agitation. Then, 50 μl of 10% trifluoroacetic acid solution was added to each sample to halt digestion. Peptides were desalted using SDB-RPS tips, according to standard protocol^86^. Samples were resuspended in 2% acetonitrile/0.3% trifluoroacetic acid, with sonication to assist solubility.

For each sample, a uniform volume (3 µl) was resolved across a 65-min gradient in data-independent acquisition mode using a Thermo PepMap 100 analytical column equipped on a Thermo Ultimate 3000 LC interfaced with a Thermo Exactive HF-X mass spectrometer. The single-injection data-independent acquisition method utilized parameters established previously^87^. Briefly, MS1 included survey scans of 1 × 10^6^ ions at a resolution of 60,000, with a maximum isolation time of 60 ms within a scan range of 390–1010 *m/z* and collision energy of 27. MS2 was performed with isolation windows of 12 *m/z* spanning a 400–1,000 *m/z* range, an automatic gain control target of 1 × 10^6^ and a resolution of 15,000.

### Other proteomics

hCOs were snap frozen on dry ice and stored at −80 °C until extraction. Pooled hCOs were lysed in 300 μl of 1% SDS in Milli-Q water with cOmplete ULTRA Protease Inhibitor Cocktail (Roche). Samples were vortexed, transferred to 2-ml Precellys Lysing Kit tubes and then pulsed three times for 30 s at 5,000 rpm with a Minilys homogenizer (Bertin Technologies). Samples were centrifuged at 8,000*g* for 10 min to remove bubbles. After centrifugation, they were transferred to microcentrifuge tubes and stored at −20 °C. Four volumes of chilled acetone were added to each sample and sonicated in 1-min bouts alternating with incubation on ice for a total of five cycles. Protein was pelleted by centrifugation at 20,000*g* for 30 min at 4 °C. Supernatant was removed, and the protein pellet was washed twice in acetone before resuspending in 1× TBS Buffer (Thermo Fisher Scientific). Reduction, alkylation, trypsin digestion (1 μg of trypsin per sample) and desalting were completed using the same method above (in DSP proteomics).

For each sample, a uniform volume (3 µl) was resolved across a 55-min gradient in data-independent acquisition mode using a Thermo PepMap 100 analytical column equipped on a Thermo Vanquish Neo UHPLC interfaced with a Thermo Exactive HF-X mass spectrometer. The single-injection data-independent acquisition method utilized parameters established previously^74^. Briefly, MS1 included survey scans of 1 × 10^6^ ions at a resolution of 60,000, with a maximum isolation time of 60 ms within the scan range of 390–1,010 *m/z* and collision energy of 27. MS2 was performed with isolation windows of 16 *m/z* spanning a scan range of 400–1,000 *m/z*, an automatic gain control target of 1 × 10^6^ and a resolution of 15,000.

### Proteomics bioinformatics

Raw spectra were analyzed using DIA-NN (version 1.8.1) against the human proteome (20,399 sequences; downloaded on 19 April 2021 from UniProt) using the library-free method. Carbamidomethylation of cysteines and N-terminal N-excision was set as a fixed modification, with matching between runs enabled. Cross-run normalization used a retention time-dependent method, and a smart-profiling method was used to generate in silico library from the human proteome.

The log_2_-transformed value for each protein was calculated for each condition and mean fold change values were generated, including *P* values (two-sided parametric *t*-test), FDR-corrected *P* values (Benjamini–Hochberg method) and *z*-scores. Principal component analysis plots were generated using R (v4.2.2) and ggfortify package (v0.4.16).

For the *CASQ2* knockout analysis, GAPDH was used as the normalization factor for CASQ2, HRC, RYR2, ATP2A2, PLN and CALR for each CASQ2 sample and the wild-type PB006.6 sample. Normalized spectral intensities for each protein in the *CASQ2* knockout samples were then expressed as the fold difference to the respective protein from the wild-type PB006.6 sample.

### Phosphoproteomics

Control hCOs or hCOs treated with 3 μM DY131 and 10 μM MK8722 or pacing at 120 bpm for 5 min were immediately quenched in ice-cold Tris-buffered saline. Sixteen hCOs were pooled per condition, and prepared for phosphoproteomics acquisition using the EasyPhos protocol^88^ with the exclusion of the C8 STAGE tip step. For each sample, a uniform volume (5 µl) was resolved across a 65-min gradient in data-dependent acquisition mode using a Thermo PepMap 100 analytical column equipped on a Thermo Ultimate 3000 LC interfaced with a Thermo Exactive HF-X mass spectrometer. The mass spectrometer performed survey scans of 3 × 10^6^ ions at a resolution of 60,000 and a range from 300 to 1,600 *m/z*. The ten most abundant precursors from the survey scan with charge state >1 and <5 were selected for fragmentation. Precursors were isolated with a window of 1.6 *m/z* and fragmented in the higher-energy collisional dissociation cell with a normalized collision energy of 27. Maximum ion fill times for the MS/MS scans were 50 ms, with a target of 2 × 10^4^ ions. Fragment ions were analyzed with high resolution (15,000) in the Orbitrap mass analyzer. Dynamic exclusion was enabled with a duration of 30 s.

### Phosphoproteomics bioinformatics

Raw liquid chromatography–mass spectrometry data were searched against the reviewed UniProt human database (20,399 sequences, downloaded 19 April 2021) using Sequest HT on the Thermo Proteome Discoverer software (version 2.3), with matching between runs enabled. Precursor and fragment mass tolerance were set to 20 ppm and 0.05 Da, respectively. A maximum of two missed cleavages were allowed. A strict FDR of 1% was used to filter peptide spectrum matches and was calculated using a decoy search. Carbamidomethylation of cysteines was set as a fixed modification, while oxidation of methionine, N-terminal acetylation and S/T/Y phosphorylation were set as dynamic modifications, with up to three phosphosites per peptide.

Differential expression analysis was performed in Proteome Discoverer software, to return log_2_ fold change values and an adjusted *P* value. Fold changes were made between electrical pacing or directed maturation conditions to control conditions. Differentially altered phosphoproteins (adjusted *P* value < 0.05) were analyzed in EnrichR^89^ using the Kyoto Encyclopedia of Genes and Genomes 2021 human gene set. All phosphoproteomics graphs were generated in GraphPad Prism, and diagrams were made in Inkscape (v1.2.2).

### Measurement of hCO respiration

Live-cell respiration was measured in real-time using Resipher (Lucid Scientific). hCOs were cultured in 96-well plates (Thermo Fisher Scientific) using Heart-Dyno inserts (height approximately 1.5 mm) that were fabricated by PDMS molding^21^. The Resipher sensor lid (9.4-mm probe length, Lucid Scientific, NS32-94A) was pre-equilibrated with naive media in a cell-free 96-well microplate at 37 °C and 5% CO_2_ overnight before seeding. After hCO formation, the Resipher sensor lid was transferred to the hCO culture plate and media O_2_ was measured with an operating height between 1,600 and 2,100 µm. During subsequent media changes, the sensor lid was stored in pre-equilibrated naive media in a cell-free microplate. On day 30 of culture, hCOs were treated with DMSO control or 10 µM BAM15 (MedChemExpress) for 6 h. Respiration rates of hCOs were normalized to cell-free control wells on the same plate, which contained naive media for each condition (SF or DM culture conditions).

### Bulk RNA-seq datasets

For maturation marker analysis, bulk RNA-seq data from published datasets were collected. To ensure cell compositions did not impact analysis, sarcomeric isoform fractions were used for the analysis including *MYH7*–*MYH6*, *MYL2*–*MYL7* and *TNNI3*–*TNNI1*. Data were collated from hPS cell-CM cultures (Gene Expression Omnibus accessions GSE93841 (ref. ^4^), GSE148025 (ref. ^7^), GSE116464 (ref. ^9^), GSE201437 (ref. ^15^), GSE114976 (ref. ^13^) and GSE151279 (ref. ^18^)) and from human hearts (ERP109940 and refs. ^19,20^).

### hCO nuclei isolation for snRNA-seq

hCOs were matured using the SF or DM protocol. At the conclusion of the experiment, the medium was aspirated and hCOs were washed with ice-cold PBS. Intact hCOs were pooled with up to 70 hCOs per sample (*n* = 2 per maturation protocol). All PBS was removed and hCOs were snap frozen in liquid nitrogen and stored at −80 °C. Nuclei isolation was completed using the 10x Chromium Nuclei Isolation Kit (PN-1000494, 10x Genomics) and 10x Genomics Chromium Nuclei Isolation Kit Protocol for Single Cell 3′ Gene Expression (RevA) with modifications. All subsequent steps in the nuclei isolation steps were performed on ice. The samples were homogenized in 200 μl lysis reagent using the pestle supplied in the 10x kit. Lysis reagent (300 μl) was added and samples were pipette mixed and left to incubate on ice for 5 min. The samples were then transferred into pre-chilled nuclei isolation columns and centrifuged at 16,000*g* for 20 s at 4 °C. Samples were vortexed for 5 s at maximum speed to resuspend nuclei and centrifuged again at 500*g* for 5 min at 4 °C. In total, 300 μl of supernatant was removed and nuclei were resuspended in 500 μl of debris removal buffer. Samples were centrifuged at 700g for 10 min at 4 °C. A total of 500 μl of supernatant was removed and nuclei were gently resuspended in 1 ml of wash and resuspension buffer. Samples were centrifuged at 500*g* for 5 min at 4 °C. Nuclei pellets were then resuspended in 250 µl wash and resuspension buffer containing DAPI (Thermo Fisher Scientific). Samples were sorted on the FACSAria III Cell Sorter using a 70-μm nozzle. Nuclei were captured in 1.5-ml microcentrifuge tubes containing 1 ml of wash and resuspension buffer. Recovered nuclei were centrifuged again at 500*g* for 5 min at 4 °C. The supernatant was removed to leave ~100 µl, and nuclei were resuspended by pipette mixing. Nuclei density counting was completed on the Countess III FL (Thermo Fisher Scientific).

Nuclei were processed using the Chromium Next GEM Single Cell 3′ GEM, Library & Gel Bead Kit v3.1 (PN-1000128, 10x Genomics). Nuclei were loaded into a Chip G (PN-1000127, 10x Genomics) and run on the Chromium Controller (10x Genomics) for gel bead emulsion (GEM) formation. Reverse transcription, barcoding, complementary DNA amplification and purification for library preparation were performed according to the Chromium Single Cell 3′ Reagent Kits User Guide (https://assets.ctfassets.net/an68im79xiti/1eX2FPdpeCgnCJtw4fj9Hx/7cb84edaa9eca04b607f9193162994de/CG000204_ChromiumNextGEMSingleCell3_v3.1_Rev_D.pdf). GEM formation and library preparation was completed by the sequencing facility at the Institute of Molecular Biosciences. All libraries were pooled and sequenced across two NovaSeq 6000 S1 flow cells (Illumina) to a depth of ~50,000 reads per nucleus (~10,000 per sample).

### snRNA-seq bioinformatics

For human fetal, young and adult hearts, snRNA-seq Fastq files were obtained from the Gene Expression Omnibus accession GSE156707 (ref. ^17^). Fastq files for human and hCO samples were aligned using 10x Cell Ranger (version 7.0). The cellranger count command with default parameters was used to align the sequencing reads to the GRCh38 build of the human transcriptome (refdata-gex-GRCh38-2020-A) and generate a gene expression count matrix.

All subsequent analysis was performed using the Seurat package in R version 4.3.0.1. There was an initial filtering step to keep genes that were expressed in three or more nuclei and nuclei with at least 200 detected genes. The quality of the cells was assessed for each sample independently by examining the total number of nuclei, the distributions of total unique molecular identifier (UMI) counts, the number of unique genes detected and the proportions of ribosomal and mitochondrial content per nuclei. Nuclei displaying high expression of mitochondrial genes (using a cutoff of <5%) were removed. For the nine human samples, nuclei with a UMI count depth of under 1,000 and higher than 40,000 were filtered out to remove debris and potential doublets. For the four hCO samples, nuclei with a UMI count depth of under 1,000 and higher than 30,000 were filtered out. No filtering was applied based on nFeature_RNA. SoupX (version 1.6.2) was used to estimate ambient RNA from empty droplets and correct the expression metrics by removing counts related to ambient RNA molecules.

For each Seurat object, transformation and normalization was performed using SCTransform to fit a negative binomial distribution and regress out mitochondrial read percentage. Integration was performed using the following functions with default parameters: SelectIntegrationFeatures, FindIntegrationAnchors and IntegrateData. Principal components were then calculated using the RunPCA function, and an elbow plot was generated to select the cutoff for significant principal components to use for downstream analysis. UMAP dimensional reduction was then computed using the top 20 principal components using the RunUMAP command. Unsupervised clustering was then performed using the FindNeighbors and FindClusters functions. For exploratory purposes, Leiden clustering with a resolution range of 0–1 increasing at increments of 0.1 was performed to identify clusters within the data.

Differential gene expression analysis was performed using the FindMarkers command with the default Wilcoxon rank-sum test. The log fold change cutoff was set to 0.25, ‘assay’ was set to ‘SCT’ and ‘slot’ was set to ‘data’. Mitochondrial genes were excluded from differential expression analysis. *P*-value adjustment was performed using Bonferroni correction based on the total number of genes in the dataset. Differentially regulated genes were determined using the cutoff of adjusted *P* value < 0.05. clusterProfiler (version 4.2.2) was used for enrichment analysis of Gene Ontology terms, using the enrichGO function. Adjusted *P* values were calculated by the Benjamini–Hochberg method and results were visualized using the dotplot function in the enrichplot R package (version 1.14.2).

### Spatial RNA-seq

hCOs were washed in PBS and evenly coated in pre-chilled Tissue-Tek O.C.T. (Sakura), then placed on a 1 × 1-cm plastic mold containing OCT compound. hCOs were arrayed in different orientations to contain six SF-hCOs and six DM-hCOs embedded in Tissue-Tek O.C.T. and frozen in an ethanol slurry. Tissue sections were sectioned at 10-μm thickness using a CM3050S cryostat (Leica), mounted onto a Stereo-seq T-Chip slide (Stereo-seq, 210CT114) pre-coated with 0.01% poly-l-lysine (Sigma) and allowed to incubate for 5 min at 37 °C. Tissue sections were then fixed in methanol for 30 min at −20 °C, and then stained with single-stranded DNA fluorescent staining solution to identify cell boundaries and imaged using Z2 Axio Imager (Zeiss) using the FITC channel. The sections were permeabilized using 1× permeabilzation reagent solution (Stereo-seq Transcriptomics T Kit, 111KT114) for 18 min at 37 °C, then rinsed in 1× PR rinse buffer.

RNA was released and reverse transcribed using Reverse Transcription Mix (Stereo-seq Transcriptomics T Kit, 111KT114) at 42 °C for 3 h. Following reverse transcription, hCOs were digested with TR Buffer (Stereo-seq Transcriptomics T Kit, 111KT114) at 55 °C for 10 min. To release cDNA from the chip, samples were incubated with cDNA Release Mix for 16 h at 55 °C. cDNA was collected, purified using 0.8× AMPure XP Beads (Beckman Coulter), and then amplified using cDNA Amplification Mix (Stereo-seq Transcriptomics T Kit, 111KT114). Samples were incubated at 95 °C for 5 min, then 15 cycles of 98 °C for 20 s, 58 °C for 20 s, 72 °C for 3 min and a final elongation step at 72 °C for 5 min.

DNA concentration was quantified using a Qubit dsDNA Assay Kit (Thermo Fisher Scientific), and cDNA fragmentation was carried out using 20 ng of cDNA and 1× Fragmentation Reaction Mix (Stereo-seq Library Preparation Kit, 111KL114) at 55 °C for 10 min. Fragmented product was amplified using PCR Barcode Primer and Amplification Mix (Stereo-seq Library Preparation Kit, 111KL114) using the following thermal cycler settings: 95 °C for 5 min, 13 cycles of 98 °C for 20 s, 58 °C for 20 s, 72 °C for 3 min and a final step at 72 °C for 5 min. The PCR product was purified using 0.55× AMPure XP Beads and sequenced using MGI DNBSEQ-T7 sequencer.

### Spatial RNA-seq bioinformatics

The stitched single-stranded DNA image was first processed through ImageStudio for compatibility with the SAW pipeline. The single Stereo-seq chip was first processed using the SAW pipeline v6.12 and aligned to the human reference genome GRCh38, using the default parameters including cellbinning. The tissue.gef output file was then analyzed using Stereopy v1.1.0. For each maturation protocol, three of the six available dynos with the highest data quality were selected and segregated into individual data objects using spatial coordinates. Each of these hCOs were then analyzed separately at a bin size of 10 and filtered for the percentage of mitochondrial genes per bin, counts per bin and genes per bin. Data were normalized for total counts and log transformed before performing Gaussian smoothing.

### Statistical analysis

An individual hCO is cultured independently and considered a biological replicate. An experiment is designated as an independent cardiac differentiation performed on an entirely different week from a different passage number of hPS cells.

For functional data, in most cases pretreatment baseline functional recordings of hCO were performed and posttreatment recordings were normalized to these as the baseline. An additional normalization to the control conditions was also performed in many cases to account for any time-dependent changes in function.

Most data were analyzed using Prism v8.2 (GraphPad) with the appropriate statistical tests depending on normality, equal variances, experimental setup and required comparisons. In general, pooled individual hCO data are skewed and experimental averages are normally distributed. Statistical tests applied and *P* values are given in each figure and legend. For omics data, data were analyzed as described in the bioinformatics sections.

### Reporting summary

Further information on research design is available in the Nature Portfolio Reporting Summary linked to this article.

## Supplementary information

Supplementary Information Supplementary Figs. 1–24.

Reporting Summary

Supplementary Tables1–9 Supplementary data and reagents.

## Source data

Source Data Fig. 1 Statistical source data.

Source Data Fig. 2 Statistical source data.

Source Data Fig. 3 Statistical source data.

Source Data Fig. 4 Statistical source data.

Source Data Fig. 5 Statistical source data.

Source Data Fig. 6 Statistical source data.

Source Data Fig. 7 Statistical source data.

Source Data Extended Data Fig. 1 Statistical source data.

Source Data Extended Data Fig. 4 Statistical source data.

Source Data Extended Data Fig. 6 Statistical source data.

Source Data Extended Data Fig. 7 Statistical source data.

Source Data Extended Data Fig. 8 Statistical source data.

Source Data Extended Data Fig. 9 Statistical source data.

## Figures and Tables

**Extended Data Table 1:**
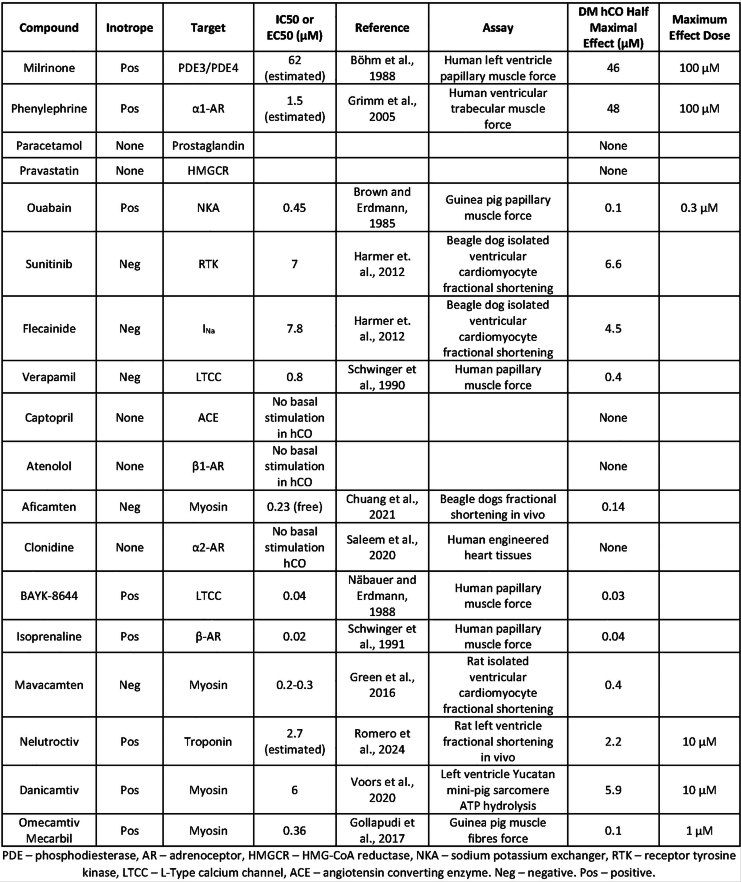
Comparison of DM-hCO drug sensitivities with *in vivo* derived heart muscle or cardiomyocytes

## Data Availability

The SF-hCO and DM-hCO snRNA-seq data are available from the Gene Expression Omnibus under accession number GSE287136. The mass spectrometry proteomics data have been deposited to the ProteomeXchange Consortium (http://proteomecentral.proteomexchange.org/) via the PRIDE partner repository^90^ under dataset identifiers PXD054810 (phosphoproteomics), PXD059940 (SF versus DM-hCO proteomics), PXD055266 (*CASQ2* knockout model) and PXD054791 (DSP cardiomyopathy model).

## Code availability

No new code was generated in this paper.
