# Supplementary material for: Maturation of human cardiac organoids enables complex disease modeling and drug discovery
Source: Nat Cardiovasc Res. 2025 Jun 25;4(7):821–40. doi: 10.1038/s44161-025-00669-3 (PMC12259470; doi:10.1038/s44161-025-00669-3)
Supplement: Supplementary file 1 — Supplementary Figs. 1–24. [file 44161_2025_669_MOESM1_ESM.pdf]

nature cardiovascular research

Supplementary information https://doi.org/10.1038/s44161-025-00669-3

Maturation of human cardiac organoids enables complex disease modeling and drug discovery

In the format provided by the authors and unedited

Table of Contents

Supplementatary Fig. 1 Increased rate in hCOs by chronic dosing of ISO

Supplementatary Fig. 2 Increased rate in hCOs using pacemaker cardiomyocytes

Supplementary Fig. 3 Increased rate in hCOs using optogenetic pacing

Supplementary Fig. 4 Increased maturation with ERR and AMPK activators

Supplementary Fig. 5 Increased maturation with progesterone, DY131 and MK8722

Supplementary Fig. 6 Interferons have differential effects on hCO function

Supplementary Fig. 7 Addition of CHIR99021 during hCO formation improves cell number

Supplementary Fig. 8 Addition of DY131 and MK8722 are optimal during the WM phase

Supplementary Fig. 9 Fatty acid type has limited impact on DM-hCO function and cTnI expression

Supplementary Fig. 10 Addition of DY131 and MK8722 improve maturation features in 2D

Supplementary Fig. 11 Phosphoproteomics on electrically paced and DM treated hCOs

Supplementary Fig. 12 Multiple cell populations are present in both SF- and DM-hCOs

Supplementary Fig. 13 Top 10 genes demarcating each cell population in SF- and DM-hCOs

Supplementary Fig. 14 UMAP projection of nuclei in SF- hCO, DM-hCOs, fetal, young and adult heart samples for cardiac fibroblast activation markers

Supplementary Fig. 15 Expression of endocardial versus coronary marker genes in SF- and DM-hCOs

Supplementary Fig. 16 Co-clustering of cardiomyocytes from SF and DM-hCOs and human heart cardiomyocytes from GSE156707

Supplementary Fig. 17 Regulated genes in specific fibroblast populations in SF- and DM-hCOs

Supplementary Fig. 18 SR and desmosomes are present in SF- and DM-hCOs

Supplementary Fig. 19 DM-hCOs predict positive inotropes at 1.8 mM Ca^{2+}

Supplementary Fig. 20 Phenotyping DSPmut mice at 45 weeks

Supplementary Fig. 21 Modelling of DSPmut in lactate-enriched 2D hPSC-CM

Supplementary Fig. 22 Fibrosis in DSPmut SF- and DM-hCOs

Supplementary Fig. 23 Proteomic analysis of DSPmut and DSPcorr DM-hCOs treated with INCB054329 (INCB)

Supplementary Fig. 24 Fibrotic and immunomodulatory signatures in proteomic analysis.

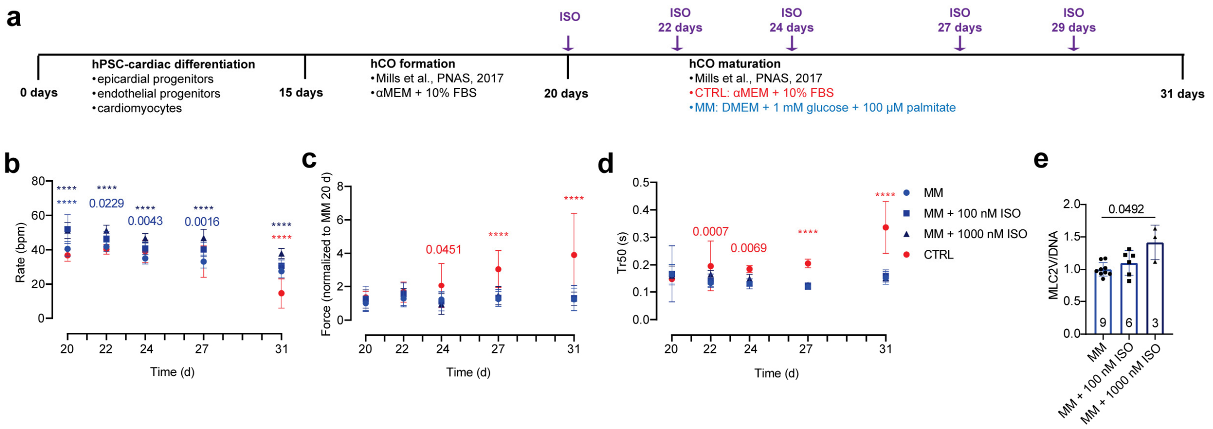

Supplementatary Fig. 1 Increased rate in hCOs by chronic dosing of ISO. a, Schematic of the protocol. **b**, Rate. **c**, Force of contraction. **d**, Time from peak to 50% relaxation (Tr50). n = hCOS and were 14-16 (MM), 9-14 (MM + 100 nM ISO), 7-14 (MM + 1000 nM ISO), 8-10 (CTRL) pooled from 2 experiments (**b-d**). **e**, MLC2V intensity normalized to DNA. n = hCOs pooled from 1-2 experiments. Data are mean ± standard deviation. Two-way ANOVA with Dunnett's multiple comparison tests to MM (**b-d**) and Kruskal-Wallis test with Dunn's multiple comparison tests to MM (**e**). **** P < 0.0001.

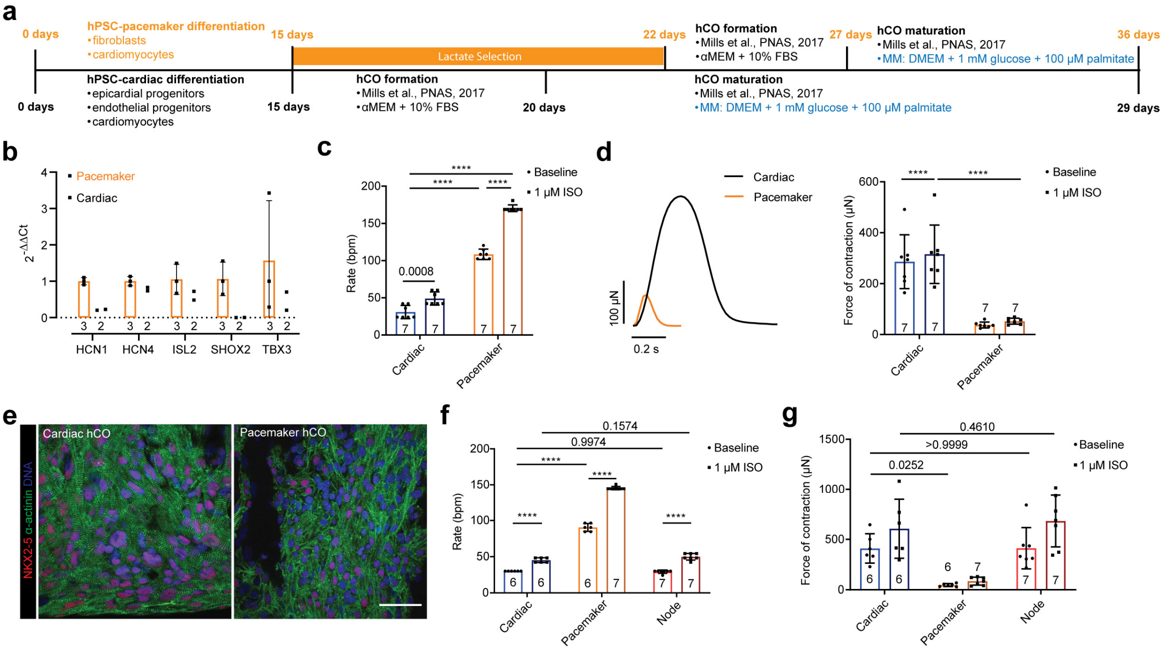

Supplementatary Fig. 2 Increased rate in hCOs using pacemaker cardiomyocytes. **a**, Schematic of the protocol. **b**, Expression of key pacemaker genes. n = 2-3 experiments. **c**, Rate. **d**, Force of contraction trace and peak force. n = hCOs (**c-d**). **e**, Expression of NKX2-5 in cardiomyocytes (α-actinin). **f**, Rate. **g**, Force of contraction. n = hCOs (**f-g**). Data are mean ± standard deviation. Two-way ANOVA with Sidaks's multiple comparison tests (**c,d,f,g**). Bar = 20 µm. **** P < 0.0001.

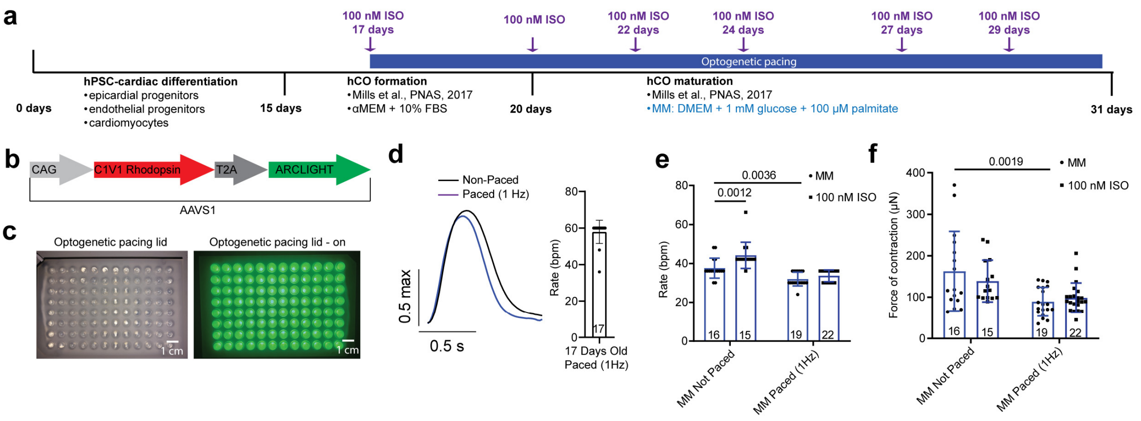

Supplementary Fig. 3 Increased rate in hCOs using optogenetic pacing. a, Schematic of the protocol. **b**, Construct inserted into AAVS1 (H9 hPSCs)**. c**, LED array lid used to stimulate pacing. **d**, Rate during pacing at the start of the protocol (17 days). n = hCOs. **e**, Rate without pacing at the end of the experiment (31 days). n = hCOs. **f**, Force of contraction. n = hCOs. Data are mean ± standard deviation. Two-way ANOVA with Sidak's multiple comparison tests (**e,f**).

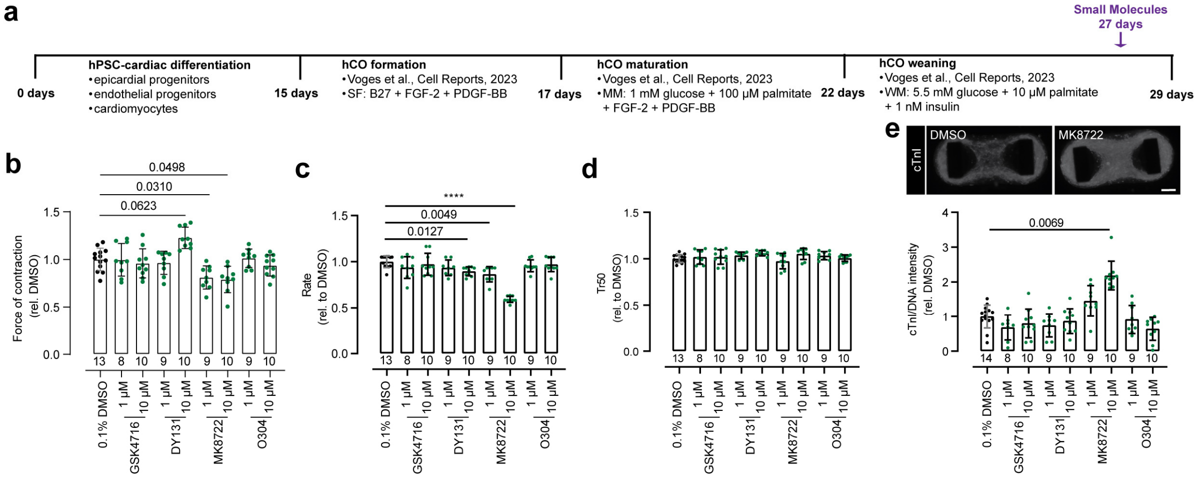

Supplementary Fig. 4 Increased maturation with ERR and AMPK activators. a, Schematic of the protocol. **b**, Force of contraction. **c**, Rate. **d**, Time from peak to 50% relaxation (Tr50). Functional data normalized to pre-treatment at 27 days. **e**, Cardiac troponin I (cTnI) intensity normalized to DNA and DMSO controls. n = hCOs pooled from 2 experiments (**b**-**e**). Data are mean ± standard deviation. Kruskal-Wallis test with Dunn's multiple comparison tests to DMSO (**b**-**e**). **** P < 0.0001. Bar = 200 µm.

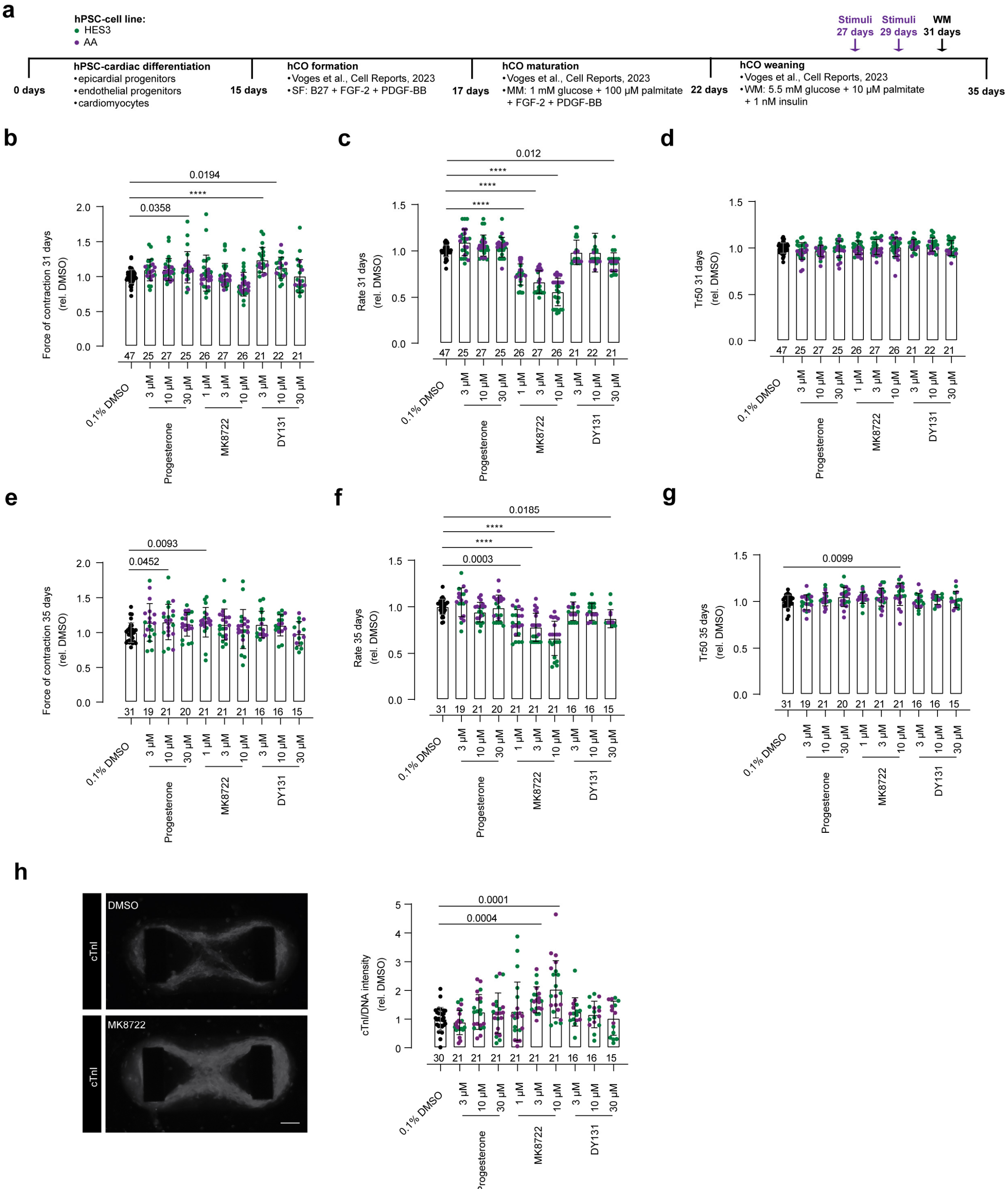

Supplementary Fig. 5 Increased maturation with progesterone, DY131 and MK8722. a, Schematic of the protocol. **b**, Force of contraction at 31 days. **c**, Rate at 31 days. **d**, Time from peak to 50% relaxation (Tr50) at 31 days. **e**, Force of contraction at 35 days. **f**, Rate at 35 days. **g**, Time from peak to 50% relaxation (Tr50) at 35 days. Functional data normalized to pre-treatment at 27 days and then to DMSO controls. **h**, Cardiac troponin I (cTnI) intensity normalized to DNA and then to DMSO controls. n = hCOs pooled from 4 experiments, 2 x HES3 (green) and 2 x AA (purple) and controls for all are in black (**b**-**h**). Data are mean ± standard deviation. Kruskal-Wallis test with Dunn's multiple comparison tests to DMSO (**b**-**h**). **** P < 0.0001. Bar = 200 µm.

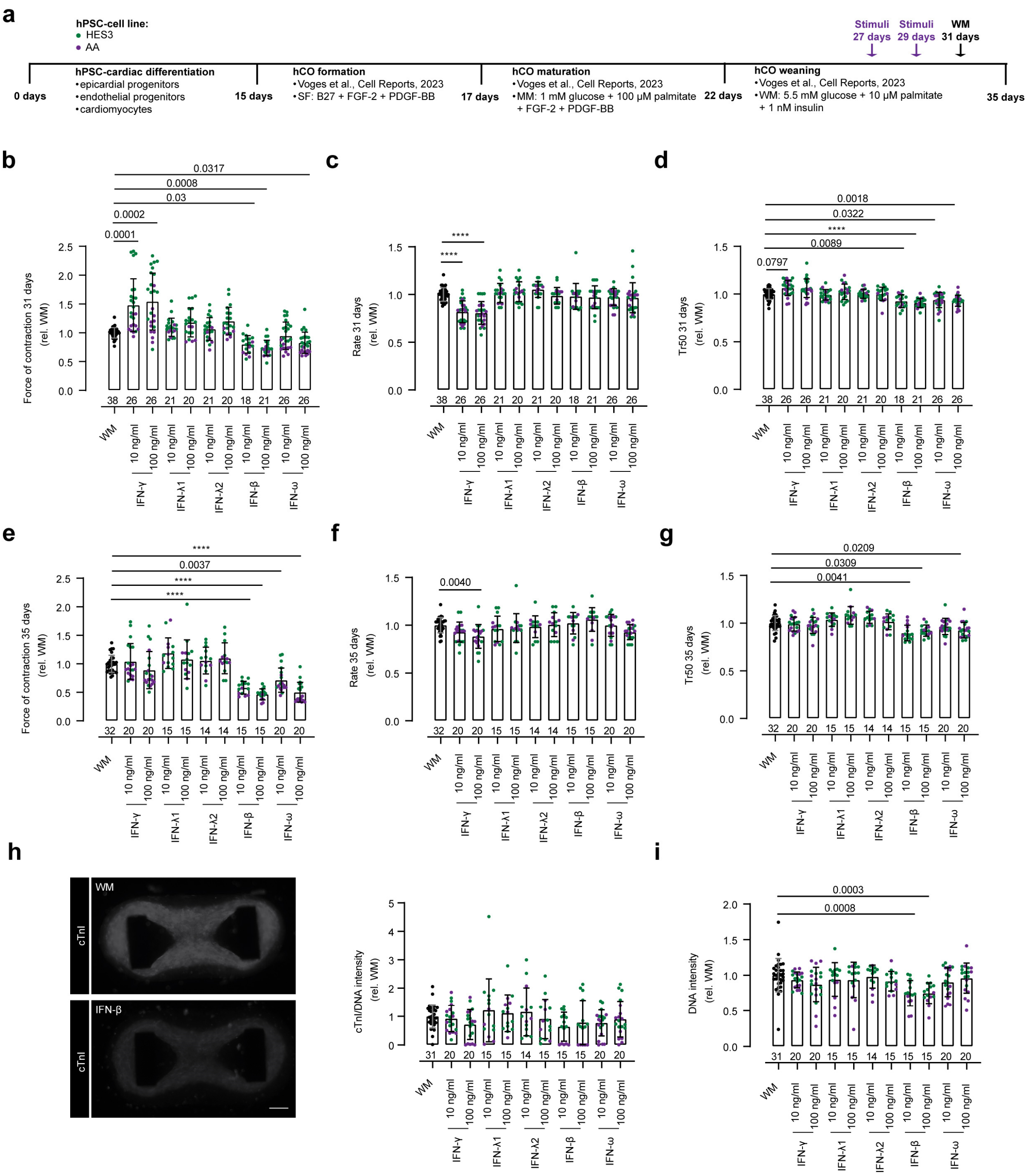

Supplementary Fig. 6 Interferons have differential effects on hCO function. a, Schematic of the protocol. **b**, Force of contraction at 31 days. **c**, Rate at 31 days. **d**, Time from peak to 50% relaxation (Tr50) at 31 days. **e**, Force of contraction at 35 days. **f**, Rate at 35 days. **g**, Time from peak to 50% relaxation (Tr50) at 35 days. Functional data normalized to pre-treatment at 27 days and then to WM controls. **h**, Cardiac troponin I (cTnI) intensity normalized to DNA and then to WM controls. **i**, DNA intensity. n = hCOs pooled from 4 experiments, 2 x HES3 (green) and 2 x AA (purple), controls for all are in black (**b-i**). Data are mean ± standard deviation. Kruskal-Wallis test with Dunn's multiple comparison tests to DMSO (**b-i**). **** P < 0.0001. Bar = 200 µm.

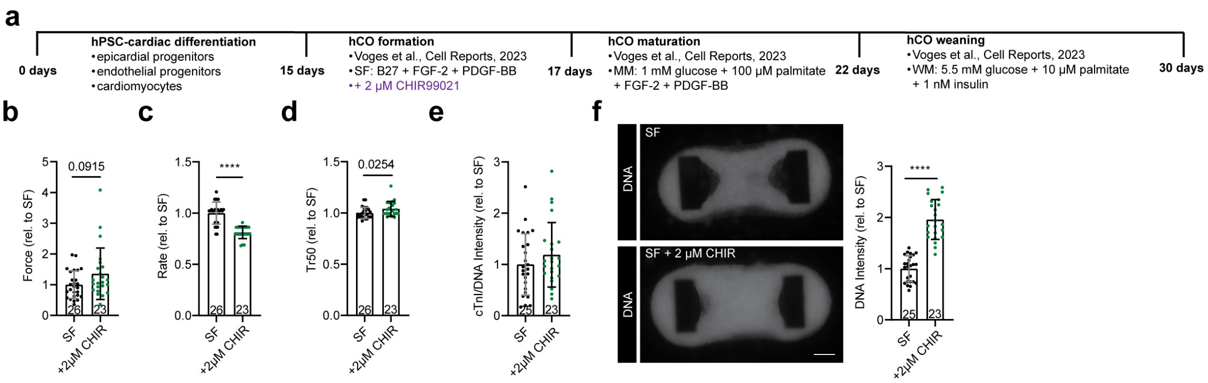

Supplementary Fig. 7 Addition of CHIR99021 during hCO formation improves cell number. **a**, Schematic of the protocol. **b**, Force of contraction at 30 days. **c**, Rate at 30 days. **d**, Time from peak to 50% relaxation (Tr50) at 30 days. Functional data normalized to the average of the SF condition for each experiment. **f**, DNA intensity. n = hCOs pooled from 2 experiments (**b**-**f**). Data are mean ± standard deviation.Two-sided Mann-Whitney test (**b**-**f**). **** P < 0.0001. Bar = 200 µm.

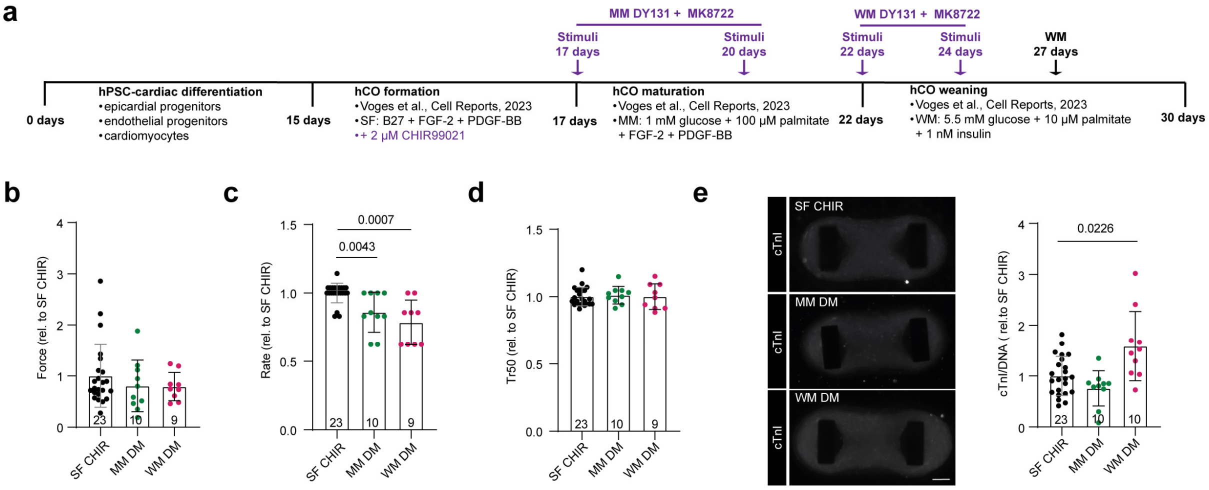

Supplementary Fig. 8 Addition of DY131 and MK8722 are optimal during the WM phase. a, Schematic of the protocol. **b**, Force of contraction at 30 days. **c**, Rate at 30 days. **d**, Time from peak to 50% relaxation (Tr50) at 30 days. **e,** Cardiac troponin I (cTnI) intensity normalized to DNA and then to SF + CHIR controls. (**b-e**). Functional data normalized to the average of the SF condition for each experiment. n = hCOs pooled from 2 experiments. Data are mean ± standard deviation. Kruskal-Wallis test with Dunn's multiple comparison tests to SF + CHIR (**b**-**f**). Bar = 200 µm.

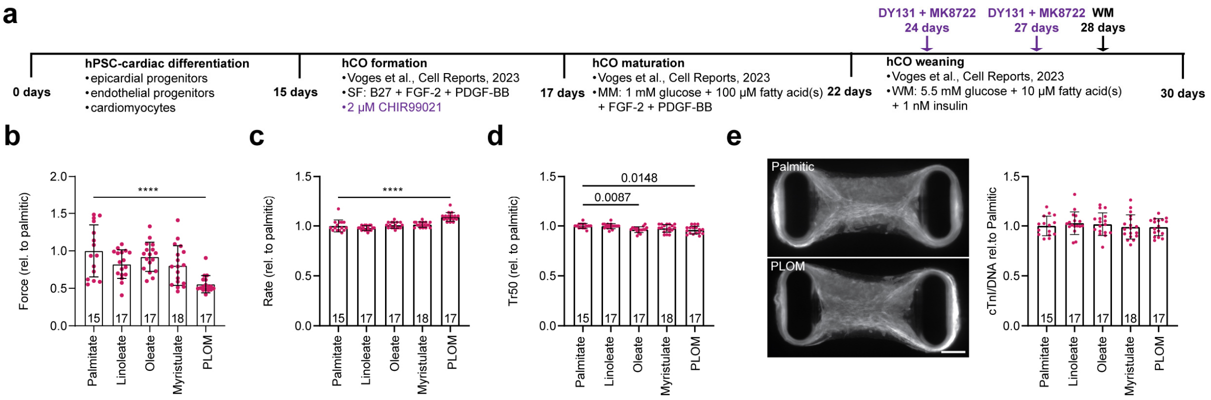

Supplementary Fig. 9 Fatty acid type has limited impact on DM-hCO function and cTnI expression. **a**, Schematic of the protocol. **b**, Force of contraction at 30 days. **c**, Rate at 30 days. **d**, Time from peak to 50% relaxation (Tr50) at 30 days. **e,** Cardiac troponin I (cTnI) intensity normalized to DNA and then to palmitate. n = hCOs pooled from 2 experiments (**b-e**). Data are mean ± standard deviation. Kruskal-Wallis test with Dunn's multiple comparison tests to palmitate (**b**-**e**). **** P < 0.0001. Bar = 200 µm. PLOM – equimolar of each fatty acid (25 µM in MM and 2.5 µM in WM).

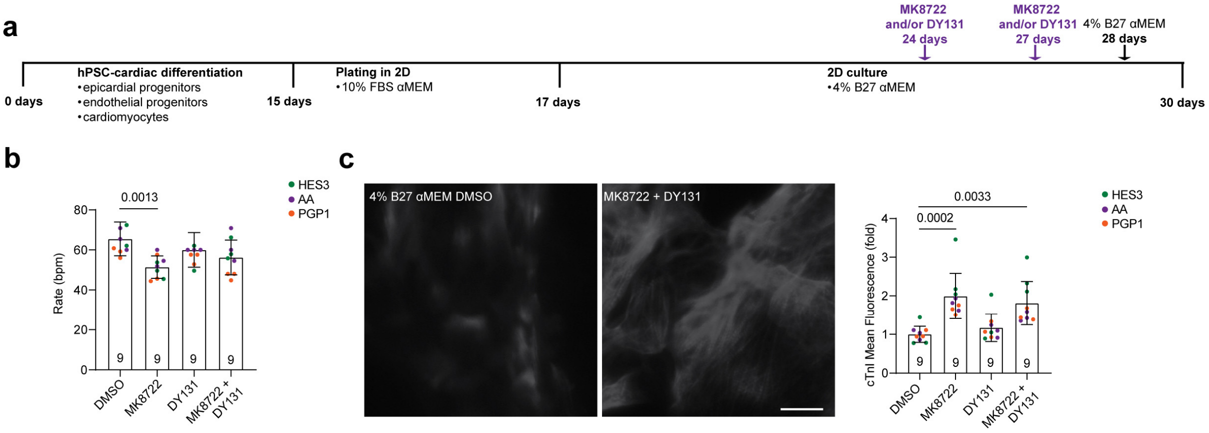

Supplementary Fig. 10 Addition of DY131 and MK8722 improve maturation features in 2D. a, Schematic of the protocol. **b**, Rate at 30 days. **c**, Normalized (to DMSO) mean fluorescence intensity (MFI) of cardiac troponin I (cTnI). n = 3 biological replicates from each cell line, HES3, AA and PGP1 which are pooled. Data are mean ± standard deviation. Kruskal-Wallis test with Dunn's multiple comparison tests to DMSO (**b**,**c**). Bar = 20 µm.

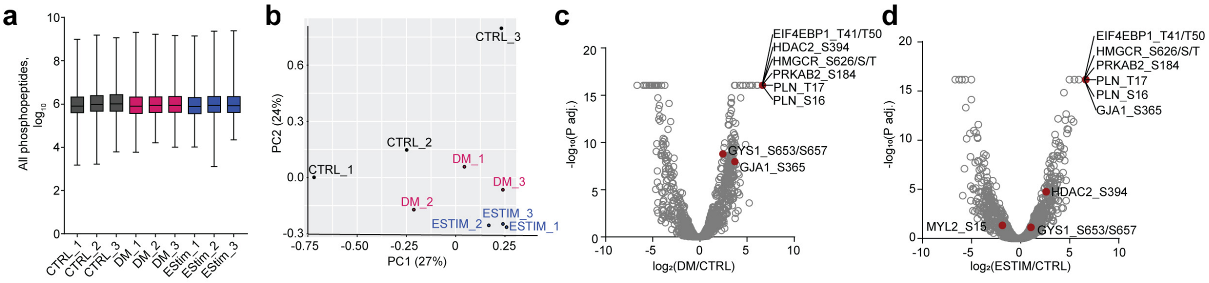

Supplementary Fig. 11 Phosphoproteomics on electrically paced and DM treated hCOs. a, Intensity of phosphopeptides across the different samples. n = 3 biological replicates per condition, each comprised of 15 pooled hCOs. **b,** Samples plotted on their principal components PC1 and PC2. **c,** Volcano plot of DM treatment versus control hCOs. **d,** Volcano plot of ESTIM versus control hCOs. DM – 10 µM MK8722 + 3 µM DY131, ESTIM – 120 bpm paced hCOs, both for 5 minutes.

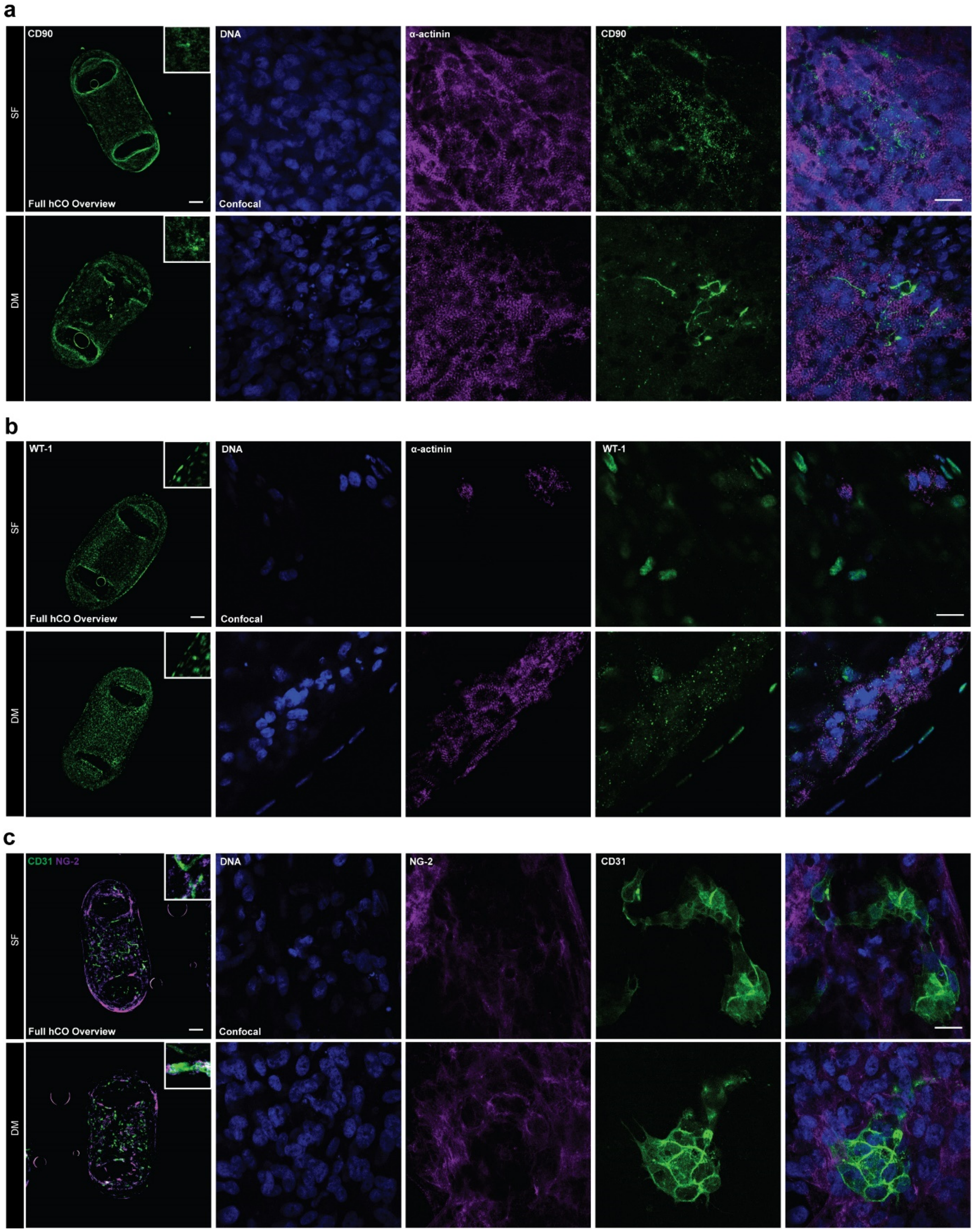

Supplementary Fig. 12 Multiple cell populations are present in both SF- and DM-hCOs. a, Fibroblast (CD90) and cardiomyocyte (α-actinin) staining. **b**, Epicardial cell (WT-1) and cardiomyocyte (α-actinin) staining. **c**, Endothelial (CD31) and pericyte (NG-2) staining. Full hCO overview bar = 200 µm. Confocal bar = 20 µm. These images are representative of hCOs from 3 different cell lines.

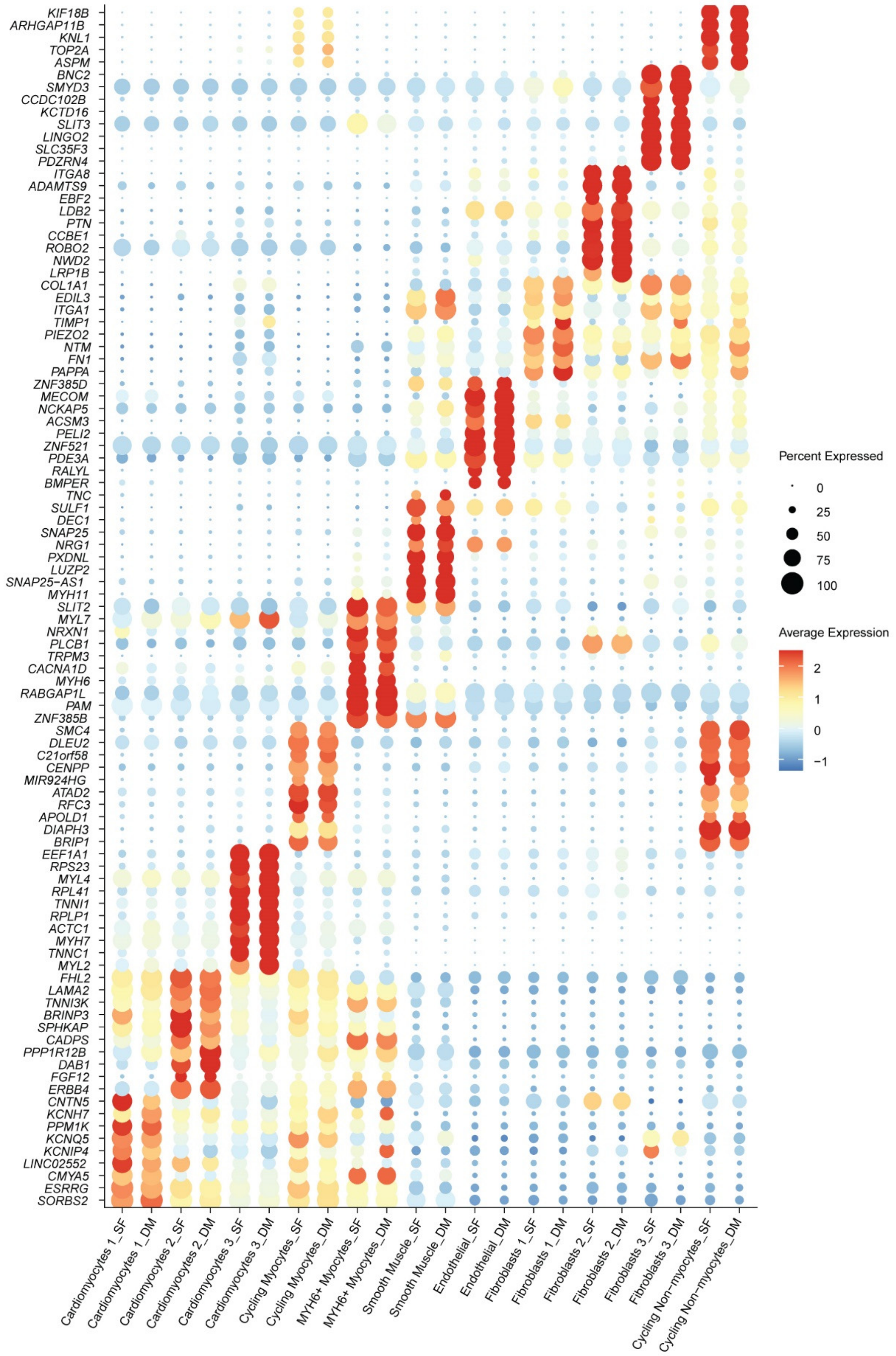

Supplementary Fig. 13 Top 10 genes demarcating each cell population in SF- and DM-hCOs.

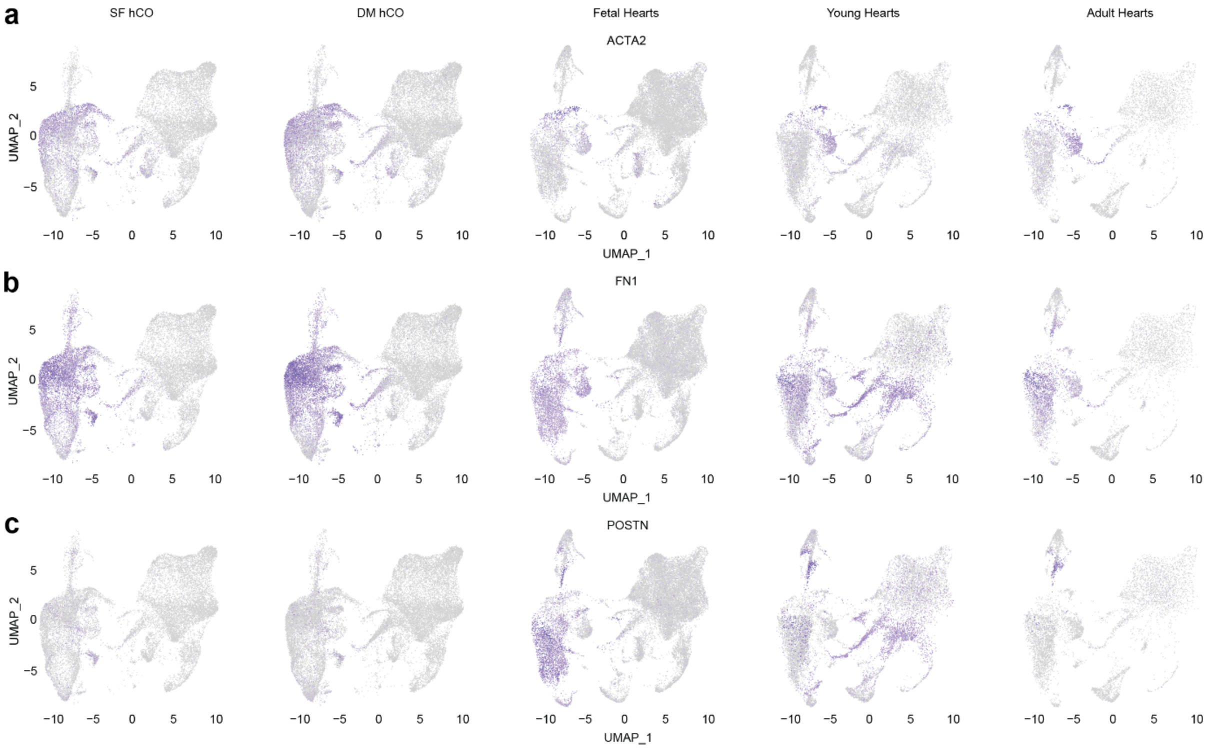

Supplementary Fig. 14 UMAP projection of nuclei in SF- hCO, DM-hCOs, fetal, young and adult heart samples for cardiac fibroblast activation markers. **a**, *ACTA2*. **b**, *FN1*. **c**, *POSTN*.

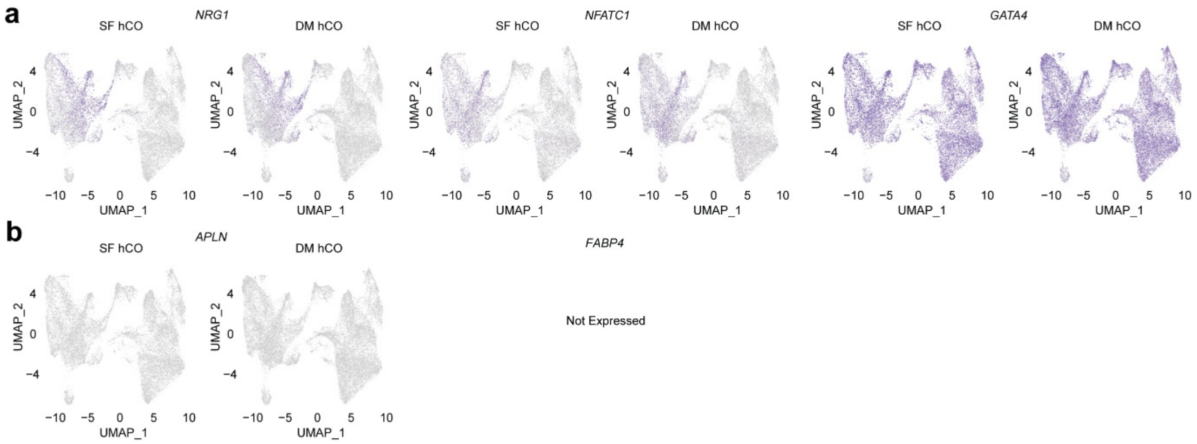

Supplementary Fig. 15 Expression of endocardial versus coronary marker genes in SF- and DM-hCOs.
a, Endocardial markers. **b**, Coronary markers.

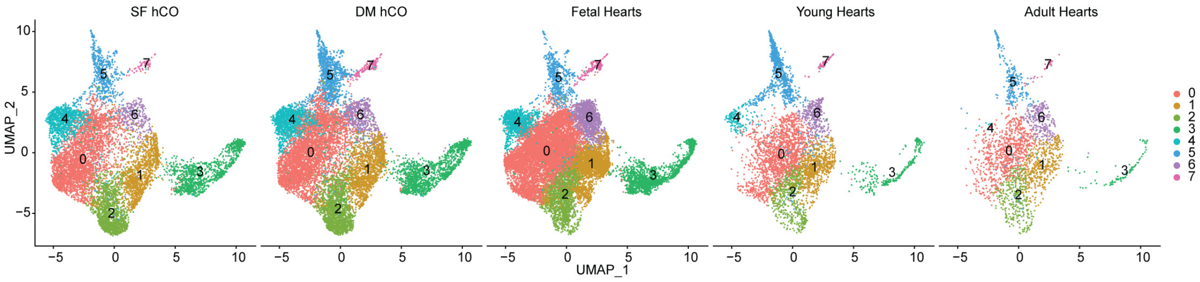

Supplementary Fig. 16 Co-clustering of cardiomyocytes from SF and DM-hCOs and human heart cardiomyocytes from GSE156707.

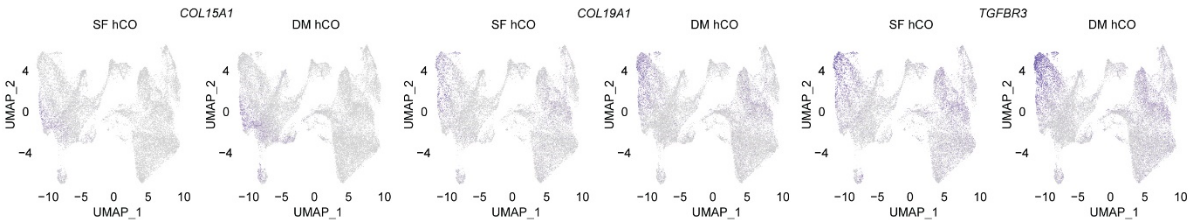

Supplementary Fig. 17 Regulated genes in specific fibroblast populations in SF- and DM-hCOs.

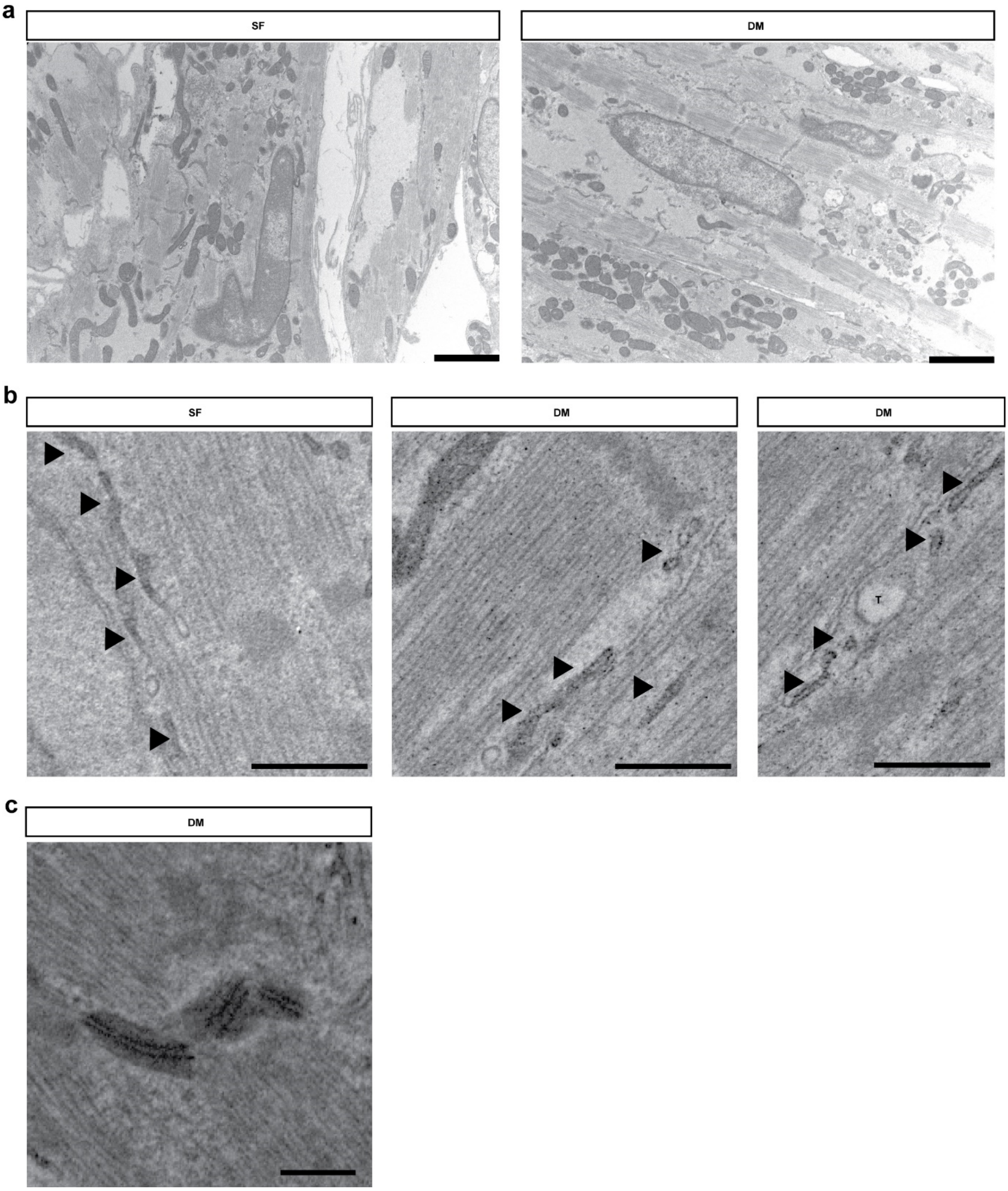

Supplementary Fig. 18 SR and desmosomes are present in SF- and DM-hCOs. a, Overview of a hCO section. Bar = 2 µm. **b**, Electron dense SR structures (indicated by arrows) and t-tubule (T). Bar = 500 nm. **c**, Electron dense intercalated discs. These images are representative of hCOs from 2 experiments. Bar = 500 nm.

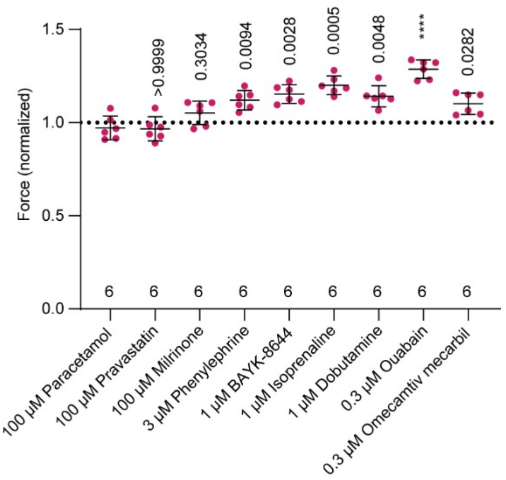

Supplementary Fig. 19 DM-hCOs predict positive inotropes at 1.8 mM Ca^{2+}. DM-hCO responses to inotropes modulating L-type calcium channel (BAYK-8644), β-adrenergic signalling (isoprenaline, dobutamine), α_1-adrenergic signalling (phenylephrine) or Na+, K+ - ATPase (ouabain), milrinone (PDE3/PDE4) and myosin (omecamtiv mecarbil). Paracetamol and pravastatin were used as inert control drugs. n = 5-6 hCOs. Data are mean ± standard deviation. Brown-Forsythe and Welch's ANOVA test with Dunnett T3 multiple comparison tests to paracetamol. **** $P < 0.0001$.

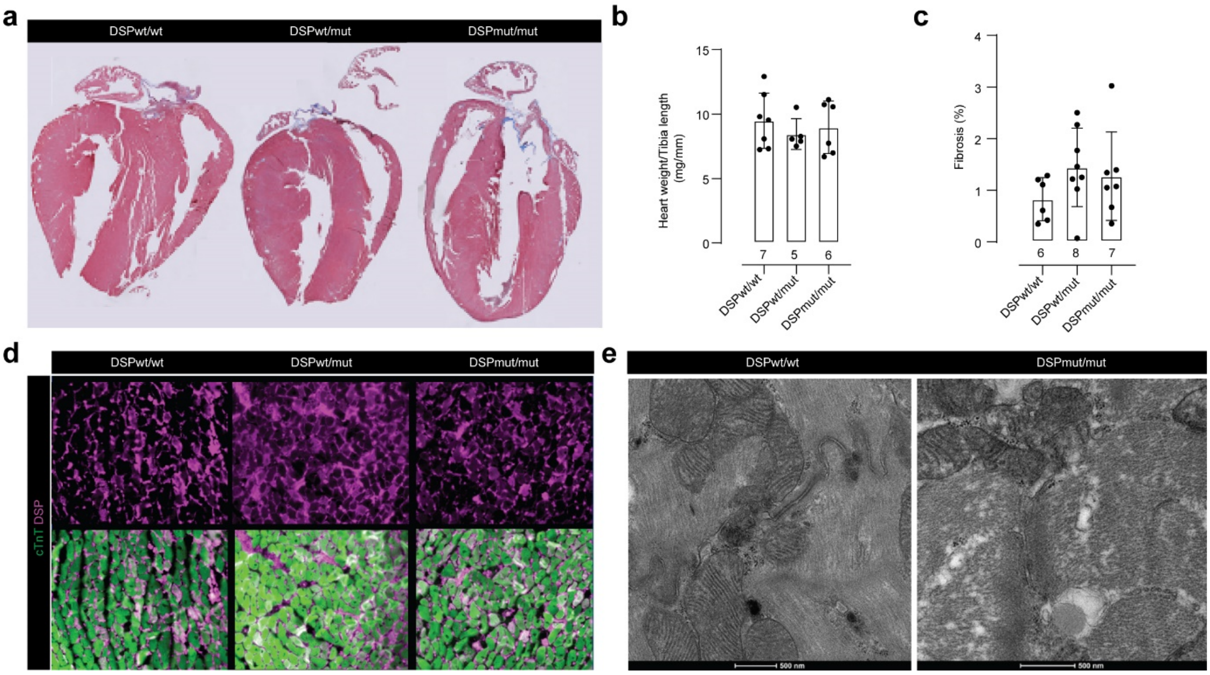

Supplementary Fig. 20 Phenotyping DSPmut mice at 45 weeks. a, Sections stained with Masson's Trichrome. **b,** Heart weight to tibia length. **c,** Analysis of fibrotic area. **d,** Immunostaining of DSP in heart sections**. e,** Transmission electron microscopy images of cell-cell junctions. Data are mean ± standard deviation. Bars = 500 nm.

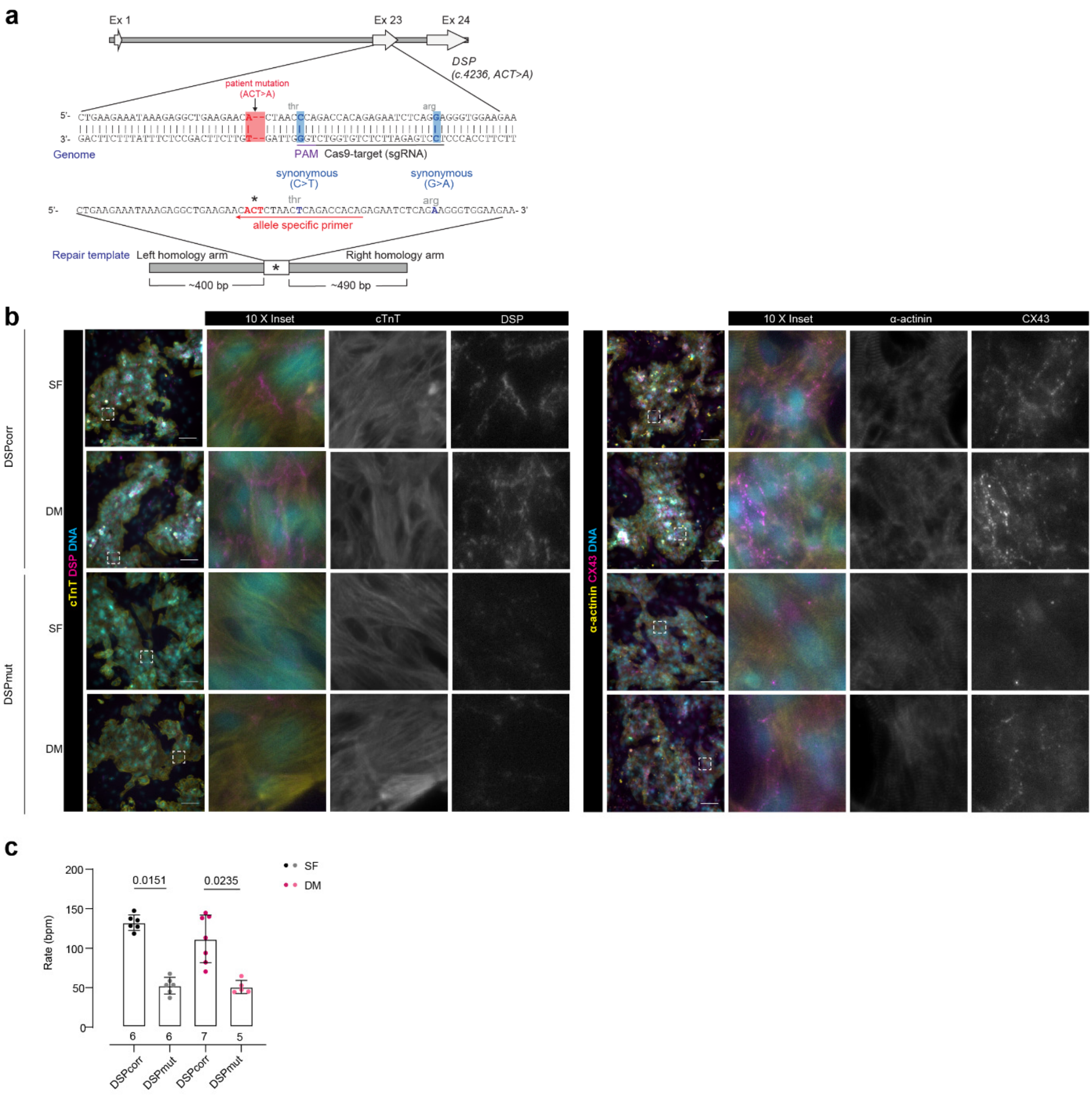

Supplementary Fig. 21 Modelling of DSPmut in lactate-enriched 2D hPSC-CM. a, Schematic of the CRISPR correction protocol**. b,** Staining for cTnT-DSP and α-actinin-CX43. Bars = 100 µm. **c**, Rate. n = biological replicates pooled from 2-4 experiments. Data are mean ± standard deviation. Kruskal-Wallis test with Dunn's post-hoc analysis (**c**). Images are representative of 2 experiments.

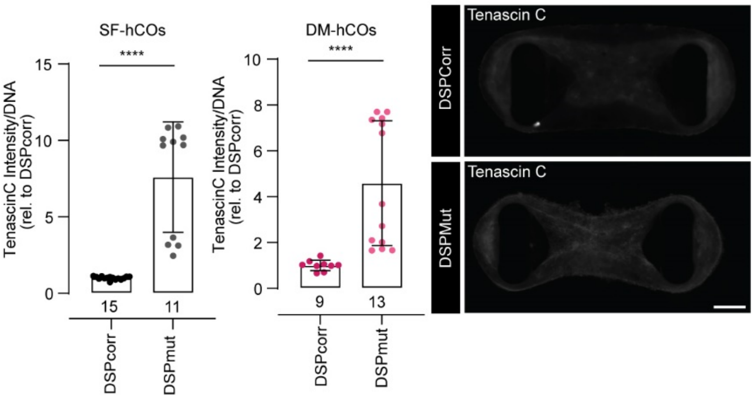

Supplementary Fig. 22 Fibrosis in DSPmut SF- and DM-hCOs. Staining for Tenascin C. n = hCOs pooled from 2 experiments. Data are mean ± standard deviation. Two-sided Mann-Whitney comparison to DSPcorr where **** indicates P < 0.0001. Bar = 200 µm.

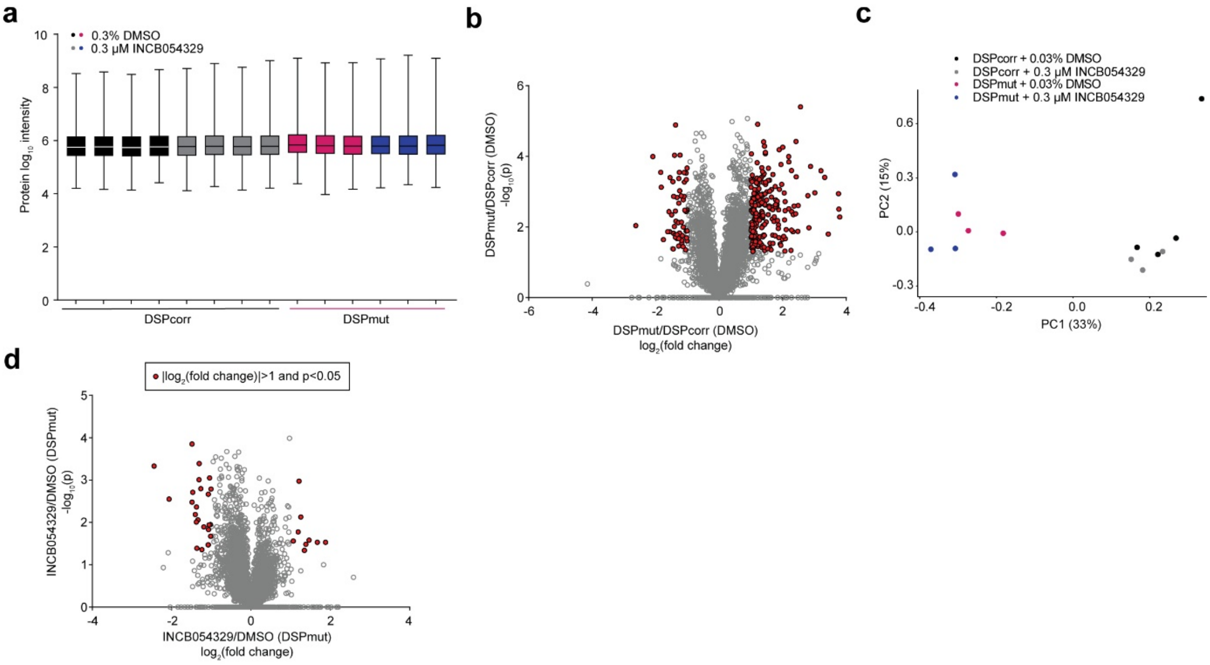

Supplementary Fig. 23 Proteomic analysis of DSPmut and DSPcorr DM-hCOs treated with INCB054329 (INCB). a, Protein abundance. n = 3-4 experiments for each condition, and each sample containing 3 pooled hCOs. **b,** Volcano plots for proteins regulated in DSPmut versus DSPcorr DM-hCOs. **c,** Principal component plot of proteomics data with treatment of INCB054329 in both DSPcorr and DSPmut DM-hCO. **d,** Volcano plots for proteins regulated by INCB054329 in DSPmut DM-hCOs.

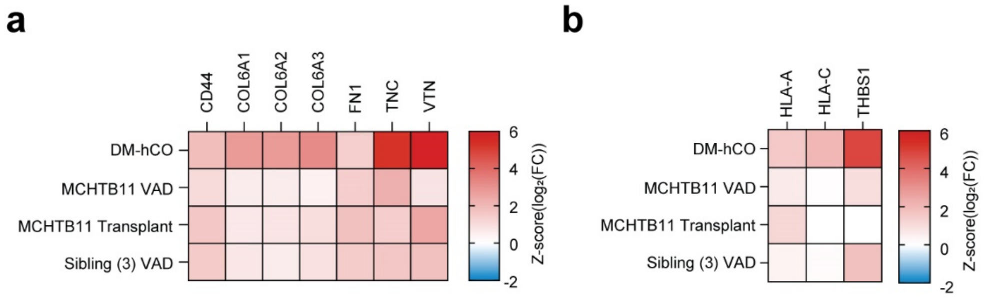

Supplementary Fig. 24 Fibrotic and immunomodulatory signatures in proteomic analysis. a, Fibrosis markers significantly upregulated in DSPmut DM-hCOs and increased in DSP mutant human heart biopsies. **b,** Immunomodulatory proteins significantly upregulated in DSPmut DM-hCOs and increased in DSP mutant human heart biopsies.
