# Supplementary material for: Maturation of human cardiac organoids enables complex disease modeling and drug discovery
Source: Nat Cardiovasc Res. 2025 Jun 25;4(7):821–40. doi: 10.1038/s44161-025-00669-3 (PMC12259470; doi:10.1038/s44161-025-00669-3)
Supplement: Supplementary file 2 — Reporting Summary [file 44161_2025_669_MOESM2_ESM.pdf]

nature portfolio

Corresponding author(s): James Hudson

Last updated by author(s): Apr 18, 2025

Reporting Summary

Nature Portfolio wishes to improve the reproducibility of the work that we publish. This form provides structure for consistency and transparency in reporting. For further information on Nature Portfolio policies, see our Editorial Policies and the Editorial Policy Checklist.

Statistics

For all statistical analyses, confirm that the following items are present in the figure legend, table legend, main text, or Methods section.

n/a	Confirmed	
☐	☒	The exact sample size (*n*) for each experimental group/condition, given as a discrete number and unit of measurement
☐	☒	A statement on whether measurements were taken from distinct samples or whether the same sample was measured repeatedly
☐	☒	The statistical test(s) used AND whether they are one- or two-sided<br>*Only common tests should be described solely by name; describe more complex techniques in the Methods section.*
☐	☒	A description of all covariates tested
☐	☒	A description of any assumptions or corrections, such as tests of normality and adjustment for multiple comparisons
☐	☒	A full description of the statistical parameters including central tendency (e.g. means) or other basic estimates (e.g. regression coefficient) AND variation (e.g. standard deviation) or associated estimates of uncertainty (e.g. confidence intervals)
☐	☒	For null hypothesis testing, the test statistic (e.g. *F*, *t*, *r*) with confidence intervals, effect sizes, degrees of freedom and *P* value noted<br>*Give P values as exact values whenever suitable.*
☒	☐	For Bayesian analysis, information on the choice of priors and Markov chain Monte Carlo settings
☒	☐	For hierarchical and complex designs, identification of the appropriate level for tests and full reporting of outcomes
☒	☐	Estimates of effect sizes (e.g. Cohen's *d*, Pearson's *r*), indicating how they were calculated

Our web collection on statistics for biologists contains articles on many of the points above.

Software and code

Policy information about availability of computer code

Data collection

Human Cardiac Organoids
We used Leica Thunder imaging with Las X software (current Version 5.3.0) to collect human cardiac organoid staked TIFFs.

Immunostaining
We used collected human cardiac organoid images at lower resolution using a Leica Thunder imaging with LasX software (current Version 5.3.0). For high resolution confocal images we used a Zeiss 780 NLO - Point scanning confocal microscope with ZEN software (Version 3.7) or a Lecia Stellaris 5 confocal microscope with Las X software (current Version 5.3.0).

Proteomics
We used Thermo Exactive HF-X mass spectrometer processed using Thermo Proteome Discoverer software (Version 2.3).

snRNA- sequencing
NovaSeq 6000 data processed using 10x Cell Ranger (version 7.0).

Spatial-sequencing
Processed using ImageStudio for compatibility with the SAW pipeline v6.12.

Data analysis

Human cardiac organoids
Human cardiac organoid stacked TIFFs were processed using Matlab available from Voges, et al., STAR Protocols, 2023 or using new commercial software Tempo.ai developed by Dynomics.

Immunostaining
Human cardiac organoid images were batch processed for intensity using Matlab (Version R2021a) or Image J (Version 1.53c).

Proteomics
Thermo Proteome Discoverer software (Version 2.3)

snRNA-sequencing
We used standard published pipelines and packages as outlined in the Methods section.

Spatial
Stereopy v1.1.0

For manuscripts utilizing custom algorithms or software that are central to the research but not yet described in published literature, software must be made available to editors and reviewers. We strongly encourage code deposition in a community repository (e.g. GitHub). See the Nature Portfolio guidelines for submitting code & software for further information.

Data

Policy information about availability of data

All manuscripts must include a data availability statement. This statement should provide the following information, where applicable:

- Accession codes, unique identifiers, or web links for publicly available datasets
- A description of any restrictions on data availability
- For clinical datasets or third party data, please ensure that the statement adheres to our policy

The SF-hCO and DM-hCO snRNA-sequencing is available from GEO with the accession number: GSE287136. Four separate proteomics data repository entries were included with this publication. The mass spectrometry proteomics data have been deposited to the ProteomeXchange Consortium (http://proteomecentral.proteomexchange.org) via the PRIDE partner repository with the dataset identifiers: PXD054810 (phosphoproteomics), PXD059940 (SF versus DM-hCO proteomics), PXD055266 (CASQ2 knockout model) and PXD054791 (DSP cardiomyopathy model).

Research involving human participants, their data, or biological material

Policy information about studies with human participants or human data. See also policy information about sex, gender (identity/presentation), and sexual orientation and race, ethnicity and racism.

Reporting on sex and gender	Details of our cell lines are provided in Supplementary Table 4 including sex.
Reporting on race, ethnicity, or other socially relevant groupings	Details of our cell lines are provided in Supplementary Table 4 including RRID so the full details can be obtained including ethnicity, age and disease status can be obtained where available.
Population characteristics	Details of our cell lines are provided in Supplementary Table 4 including age.
Recruitment	Patients with cardiomyopathy were recruited as part of the Melbourne Children's Heart Tissue Bank (MCHTB) program under informed consent.
Ethics oversight	Ethical approval for the generation and/or use of human heart tissue and hPSCs were obtained from QIMR Berghofer's Ethics Committee and Murdoch Children's Research Institute (MCRI) Ethics Committees and were carried out in accordance with the National Health and Medical Research Council of Australia regulations. Informed consent was obtained from all participants. hPSCs were obtained from WiCell, Coriell, MCRI and the CIRM hPSC Repository funded by the California Institute of Regenerative Medicine as detailed in the Supplementary Table 4. hPSC cell lines are available upon request with appropriate agreements and ethical approvals.

Note that full information on the approval of the study protocol must also be provided in the manuscript.

Field-specific reporting

Please select the one below that is the best fit for your research. If you are not sure, read the appropriate sections before making your selection.

☒ Life sciences ☐ Behavioural & social sciences ☐ Ecological, evolutionary & environmental sciences

For a reference copy of the document with all sections, see nature.com/documents/nr-reporting-summary-flat.pdf

Life sciences study design

All studies must disclose on these points even when the disclosure is negative.

Sample size	Using our previous screening data as a foundation (Mills et al., Cell Stem Cell, 2029 and Mills et al., Cell, 2021), n = 4-6 human cardiac organoids per condition is sufficient to detect functional changes >10%. Then experiments need to be repeated multiple times to ensure reproducibility.
Data exclusions	There is cardiac differentiation QC for contractile cardiomyocytes ~70% and functioning human cardiac organoids typically producing forces of >30 uN at baseline prior experiments.<br><br>In the proteomics data in Fig. 7e some samples with very low or undetected protein abundance needed to be removed as they were outliers.
Replication	All numerical replication data is presented. Where an experiment was very important for the conclusions of the paper it was repeated at least 3 times and in multiple cell lines as described in the figure legends. The exceptions were some proteomics data and the snRNA-sequencing data as these can be very considerable experimental undertakings - see figure legends for each experiment.
Randomization	Human cardiac organoids are randomized into groups prior to experimentation.
Blinding	The functional data and immunostaining analysis pipeline is automated and agnostic to the conditions. Researchers were not blinded to group allocation after data was processed as some experiments have very complicated timelines and plate layouts, requiring knowledge of experimental conditions in order to analyze and normalize the data.

Reporting for specific materials, systems and methods

We require information from authors about some types of materials, experimental systems and methods used in many studies. Here, indicate whether each material, system or method listed is relevant to your study. If you are not sure if a list item applies to your research, read the appropriate section before selecting a response.

Materials & experimental systems

n/a	Involved in the study	
☐	☒	Antibodies
☐	☒	Eukaryotic cell lines
☒	☐	Palaeontology and archaeology
☐	☒	Animals and other organisms
☒	☐	Clinical data
☒	☐	Dual use research of concern
☒	☐	Plants

Methods

n/a	Involved in the study	
☒	☐	ChIP-seq
☒	☐	Flow cytometry
☒	☐	MRI-based neuroimaging

Antibodies

Antibodies used	These are all are described and detailed in Supplementary Table 9.<br><br>Mouse IgG1 anti-Desmoplakin I+II antibody, Abcam, Cat no. ab16434, Clone DP2.15<br>Mouse IgG1 anti-α-actinin, Sigma, Cat no. A7811, Clone EA-53<br>Rabbit anti-cardiac troponin T, Abcam, Cat no. ab45932<br>Mouse IgG1 anti-cardiac troponin T antibody, Abcam, Cat no. ab8295, Clone 1C11<br>Mouse IgG2A anti-Neural/Glial Antigen 2, Thermo Fisher Scientific, Cat no. 14-6504-82, Clone 9.2.27<br>Rabbit anti-Wilms Tumor Protein (WT-1), Abcam, Cat no. ab89901, Clone CAN-R9(IHC)-56-2<br>Mouse IgG2A anti-CD90/Thy1, R&D Systems, Cat no. MAB2067, Clone Thy-1A1<br>Mouse IgG1 anti-human CD31, Dako, Cat no. M0823, Clone JC70A<br>Mouse IgG1 anti-desmoplakin, Progen, Cat no. 65146, Clone DP1&2-2.15; DP1-2.17; DP1-2.20<br>Rabbit anti-connexin 43, Abcam, Cat no. ab11370<br>Chicken IgY anti-GFP, Abcam, Cat no. ab13970<br>Mouse IgG2A anti-cardiac troponin I, Abcam, Cat no. ab10231, Clone 4C2<br>Goat anti-Mouse IgG (H+L) Cross-Adsorbed Secondary Antibody, Alexa Fluor 488, Thermo Fisher Scientific, Cat no. A-11001<br>Goat anti-Mouse IgG (H+L) Cross-Adsorbed Secondary Antibody, Alexa Fluor 555, Thermo Fisher Scientific, Cat no. A-21422<br>Goat anti-Rabbit IgG (H+L) Cross-Adsorbed Secondary Antibody, Alexa Fluor 488, Thermo Fisher Scientific, Cat no. A-11008<br>Goat anti-Rabbit IgG (H+L) Cross-Adsorbed Secondary Antibody, Alexa Fluor 555, Thermo Fisher Scientific, Cat no. A-21428
Validation	Mouse IgG1 anti-Desmoplakin I+II antibody, Abcam, Cat no. ab16434<br>Manufacturer reports expected reactivity in human samples for ICC, WB, ICC/IF, and IHC-Fr. Manufacturer provides 33 citations using the antibody with human and mouse samples and ICC/IF.<br><br>Mouse IgG1 anti-α-actinin, Sigma, Cat no. A7811

From manufacturer's website: Validated for IHC by "Staining of formalin-fixed, paraffin-embedded section of human tongue using Monoclonal Anti-α-Actininn (Cat. No. A7811) showing specific staining of striated muscle. Antibody dilution 1:800."

Rabbit anti-cardiac troponin T, Abcam, Cat no. ab45932
From manufacturer's website: "Anti-Cardiac Troponin T antibody (ab45932) is a rabbit polyclonal antibody and is validated for use in IHC-P, WB, sELISA in human, mouse, rat samples... Anti-Cardiac Troponin T antibody (ab45932) specifically detects Cardiac Troponin T (UniProt ID: P45379; Molecular weight: 36kDa) and is sold in 100 μg selling sizes."

Mouse IgG1 anti-cardiac troponin T antibody, Abcam, Cat no. ab8295
From manufacturer's website: "Anti-Cardiac Troponin T antibody [1C11] (ab8295) is a mouse monoclonal antibody and is validated for use in ICC/IF, IHC-P and sELISA. Anti-Cardiac Troponin T antibody [1C11] (ab8295) specifically detects Cardiac Troponin T (UniProt ID: P45379; Molecular weight: 36kDa)..."

Mouse IgG2A anti-Neural/Glial Antigen 2, Thermo Fisher Scientific, Cat no. 14-6504-82
From manufacturer's website: Validated for ICC/IF on human A-375 cells.

Rabbit anti-Wilms Tumor Protein (WT-1), Abcam, Cat no. ab89901
From manufacturer's website: "Anti-Wilms Tumor Protein antibody [CAN-R9(IHC)-56-2] (ab89901) was developed by Abcam using patented rabbit monoclonal antibody technology and is validated for use in Flow Cyt (Intra), ICC/IF, IHC-P and WB." "Anti-Wilms Tumor Protein antibody [CAN-R9(IHC)-56-2] (ab89901) specifically detects Wilms Tumor Protein (UniProt ID: P19544; Molecular weight: 50kDa) and is sold in 100 μL and 1 mL selling sizes."

Mouse IgG2A anti-CD90/Thy1, R&D Systems, Cat no. MAB2067
Manufacturer provides 8 citations, including demonstrations of the use to the product in human samples for IHC: Wei, K., Korsunsky, I., Marshall, J.L. et al. Notch signalling drives synovial fibroblast identity and arthritis pathology. Nature 582, 259–264 (2020). https://doi.org/10.1038/s41586-020-2222-z

Mouse IgG1 anti-human CD31, Dako, Cat no. M0823
From the manufacturer's website: "The antibody was clustered as anti-CD31 at the Fifth International Workshop and Conference on Human Leucocyte Differentiation Antigens. The epitope recognized was found to be within the extracellular domain 1.
In Western blotting of membrane preparations from a spleen rich in the antigen or from normal platelets, the antibody labels bands of respectively 100 kDa and 130 kDa, the latter corresponding to classic CD31. The smaller band of 100 kDa observed with the splenic preparation may be due to proteolytic breakdown or to variations in glycosylation."

Mouse IgG1 anti-desmoplakin, Progen, Cat no. 65146
From manufacturer's website: "Tested cultured cell lines: several human carcinoma cell lines: A-431, BMGE, MDCK."

Rabbit anti-connexin 43, Abcam, Cat no. ab11370
Manufacturer states antibody tested on human tissue for IHC-P and WB. Manufacturer cites customer review with accompanying image: "ab11370 staining Connexin 43 / GJA1 in human pluripotent stem cell derived cardiomyocyte by ICC/IF (Immunocytochemistry/immunofluorescence)."

Chicken IgY anti-GFP, Abcam, Cat no. ab13970
Manufacturer states antibody tested for WB and ICC/FF. Manufacturer provides citation metrics: antibody has been cited in 3612 publications.

Mouse IgG2A anti-cardiac troponin I, Abcam, Cat no. ab10231
Manufacturer states antibody tested for human tissue for IHC-P. Manufacturer provides citation metrics: antibody has been cited in 25 publications.

Eukaryotic cell lines

Policy information about cell lines and Sex and Gender in Research

Cell line source(s)	These are all described in Supplementary Table 4 including RRID.
Authentication	We authenticated the lines using DNA fingerprinting and test karyotypes for cell line stability.
Mycoplasma contamination	We regularly test for mycoplasma and had no positive instances over the course of this study.
Commonly misidentified lines (See ICLAC register)	No commonly misidentified lines were used in this study.

Animals and other research organisms

Policy information about studies involving animals; ARRIVE guidelines recommended for reporting animal research, and Sex and Gender in Research

Laboratory animals	DSPrul (RB156Bnr/Ei-Dsprul/GrsrJ) mice were obtained from The Jackson laboratory (Strain #005362). Mice were assessed at 45 weeks of age.
Wild animals	No wild animals were used in this study.

Reporting on sex	We initially segregated the male and female data, but there were no differences between controls and the DSP mutant mice for either sex so all data were combined (heart weight to tibia length and fibrosis).
Field-collected samples	No field collected samples were used in this study.
Ethics oversight	Animal experiments were conducted in accordance with the relevant codes of practice for the care and use of animals for scientific purposes as stipulated by the National Health and Medical Research Council of Australia and conducted with approval from the Animal Ethics Committee at the Murdoch Children's Research Institute (A958).

Note that full information on the approval of the study protocol must also be provided in the manuscript.

Plants

Seed stocks	*Report on the source of all seed stocks or other plant material used. If applicable, state the seed stock centre and catalogue number. If plant specimens were collected from the field, describe the collection location, date and sampling procedures.*
Novel plant genotypes	*Describe the methods by which all novel plant genotypes were produced. This includes those generated by transgenic approaches, gene editing, chemical/radiation-based mutagenesis and hybridization. For transgenic lines, describe the transformation method, the number of independent lines analyzed and the generation upon which experiments were performed. For gene-edited lines, describe the editor used, the endogenous sequence targeted for editing, the targeting guide RNA sequence (if applicable) and how the editor was applied.*
Authentication	*Describe any authentication procedures for each seed stock used or novel genotype generated. Describe any experiments used to assess the effect of a mutation and, where applicable, how potential secondary effects (e.g. second site T-DNA insertions, mosiacism, off-target gene editing) were examined.*
